# Complex eruption processes and deposits of basaltic fissures: insights from the ~37 ka Budj Bim volcanic complex, Southeastern Australia

**DOI:** 10.1007/s00445-026-01967-9

**Published:** 2026-03-31

**Authors:** Janine L. Kavanagh, Katy J. Chamberlain, Jade A. Hrintchuk, Elisabetta Mariani, Julie Boyce, Stefano Urbani, Tegan A. Havard, Kate M. Williams, Ray A. F. Cas

**Affiliations:** 1 School of Environmental Sciences, Jane Herdman Laboratories, University of Liverpool, Liverpool, L69 3GP UK; 2 School of Earth, Atmosphere and Environment, Monash University, Clayton, VIC 3800 Australia; 3 Volcanoes Discovery Centre, Penshurst, VIC 3294 Australia; 4 Department of Geological Survey of Italy, Institute for Environmental Protection and Research (ISPRA), Via Vitaliano Brancati 48, 00100 Rome, Italy; 5 Centrum Badań Kosmicznych Polskiej Akademii Nauk (CBK PAN), Warsaw, Poland; 6 School of Earth and Environment, Leeds University, Leeds, LS2 9JT UK

**Keywords:** Basalt, Scoria cone, Lava flow, Feeder dyke, Xenopumice, Fault zone

## Abstract

Throughout Earth’s history, eruptions from volcanic fissures have been responsible for some of the largest outpourings of basaltic magma, impacting climate and society. In continental monogenetic settings, these eruptions can occur on short timescales and with short notice after long quiescence. Their deposits are often quickly destroyed or buried as the eruption progresses, limiting understanding of eruption triggers and hazards uniquely garnered from these tephra layers and lavas. The ~37 ka Budj Bim Volcanic Complex (BBVC) in the active intracontinental monogenetic Newer Volcanics Province (NVP), southeastern Australia (~4.5 Ma to present), is a rarely preserved volcanic fissure system that provides new insight on how magma ascent dynamics impact eruptions. The BBVC comprises a 2.2 km long array of basaltic scoria cones, spatter cones, an elongate open crater with steep inward-sloping walls and a substantial lava-tube-fed flow field. Newly logged eruption sequences, petrography and geochemical analysis reveal five eruption stages, involving vent-shifting along a dyke-fed fissure, and rapid changes in eruption styles between Strombolian and micro-Plinian activity interspersed with concurrent and intermittent phreatomagmatism. The eruption culminated in the establishment and then breach of an elongate lava lake, feeding the > 30 km long culturally significant Tyrendarra lava flow (UNESCO World Heritage site). The BBVC eruption would have evolved similarly to the 2014–2015 Bárðarbunga-Holuhraun fissure eruption in Iceland, but with increased complexity due to its intraplate continental setting and feeder dyke interaction with aquifers and near-surface faults, resulting in greater level of phreatomagmatism and explosion depths potentially down to 320 m. This finding must be considered to improve future eruption scenario assessments in the NVP and builds new understanding of complex volcanic fissure eruption behaviour that can be applied to similar systems around the world.

**Supplementary Information:**

The online version contains supplementary material available at 10.1007/s00445-026-01967-9.

## Introduction

**Fig. 1:**
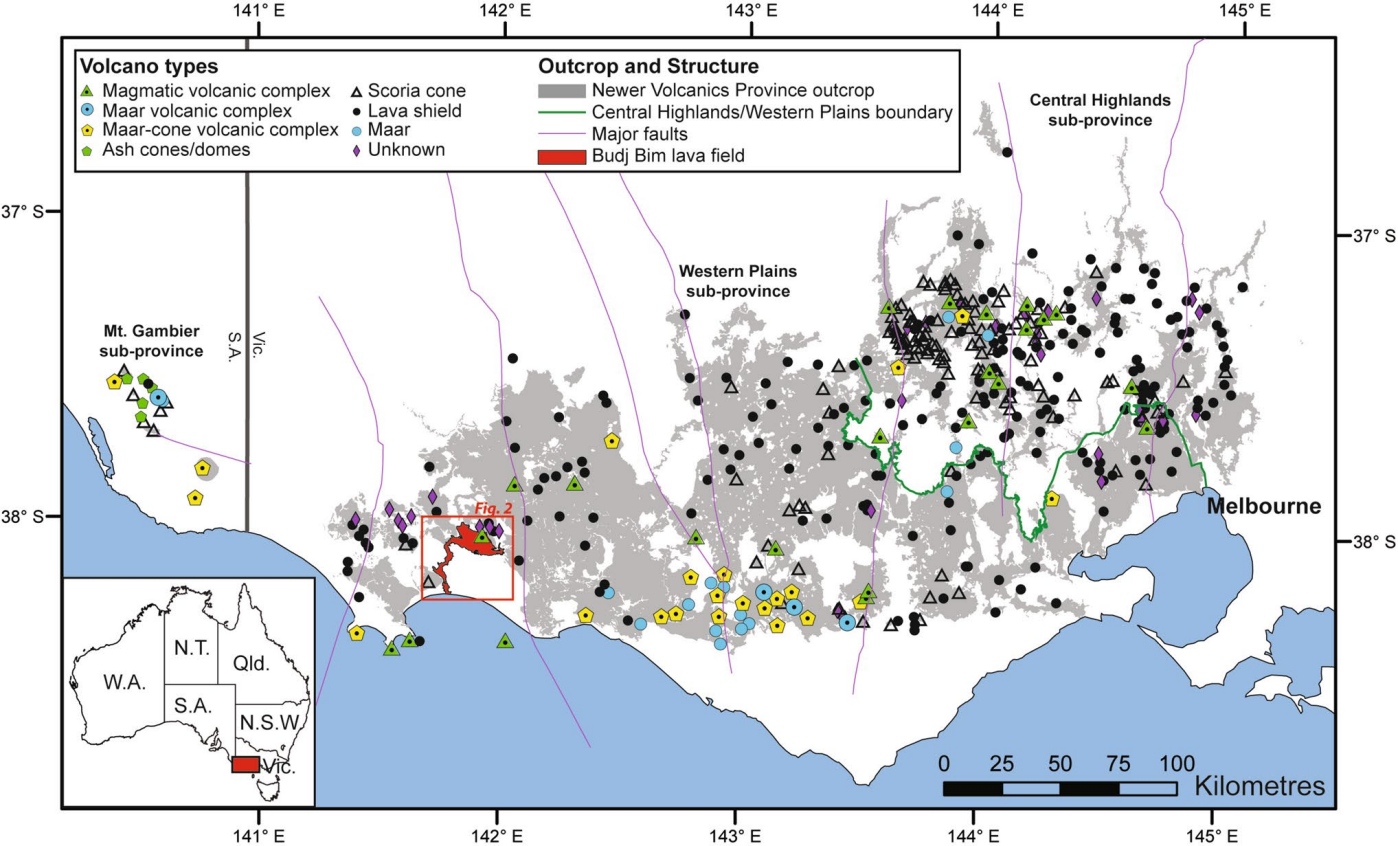
Volcanoes of the Newer Volcanic Province in southeastern Australia (modified after Boyce (2013), see inset map for regional location). Red box indicates the zoomed-in area of the BBVC (38°3.66′ S, 141°55.34′ E) shown in Fig. 2

The Newer Volcanics Province (NVP, 8 Ma–present (Gray and McDougall 2009; Cas et al. 2017)) is the most recent site of volcanic activity in Australia. This very large intraplate intracontinental monogenetic basaltic province covers an area of > 19,000 km^2^ as mapped geologically (Boyce 2013), extends 410 km from Melbourne, Victoria to Mount Burr, South Australia, and has a total erupted volume of up to 900 km^3^ (dense rock equivalent) (Lesti et al. 2008; Cas et al. 2017). Eruptions from more than 400 mostly monogenetic volcanoes encompass both simple lava shields, scoria cones, maars, ash cones and domes, with complex magmatic/phreatomagmatic centres, multiple eruption points and complex morphologies (Fig. 1; Boyce (2013)). Eruptions would typically have been small volume and short-lived (Smith and Németh 2017), lasting hours to days, weeks to months or rarely a few years (Blaikie et al. 2015; Cas et al. 2017). Even small volume eruptions in the future would potentially be very disruptive to the local area and to Melbourne (population > 5.3 million; Australian Bureau of Statistics 2023–24, 2025); road closures from lava flows (e.g. Tsang and Lindsay 2020; Hayes et al. 2022), volcanic ash accumulating on roofs of nearby buildings and infiltrating infrastructure such as water supplies (Wilson et al. 2012), respiratory problems due to inhalation of ash (Horwell and Baxter 2006) and the potential closure of airports or disruption to air traffic (Cas et al. 2017; Delbrel et al. 2025) are all potential impacts of a new volcanic event in the NVP (Handley et al. 2018). It is therefore important to constrain the eruptive history of volcanoes in the NVP to understand the style, hazards and risks associated with future eruptions.

Fissure vents are elongate fractures through which magma exits to feed often simultaneous explosive eruptions and lava effusions (Hargitai and Kereszturi 2015). Their orientation reflects the surface expression of a subsurface dyke, which in a monogenetic volcanic field can take on a range of orientations due to stress field variations over time and complexities in crustal architecture, including faults (Runge et al. 2014; Németh et al. 2024). Since 2014, significant basaltic fissure eruptions around the world have occurred in locations such as Iceland due to tectonic rifting (e.g. 2014–2015 (Pedersen et al. 2017), 2021–present (Hjartardóttir et al. 2023)) and Hawaiʻi along rift zones in an intraplate setting (e.g. 2018; Houghton et al. (2021)), providing new observations and datasets (Taddeucci et al. 2015). Eruptive activity will often begin with a curtain of lava fountaining along the length of the crack, potentially sustaining an elongate lava lake and building a spatter rampart (e.g. Pedersen et al. 2017). Activity may migrate along the vent over time and/or localise to a few discrete points, forming lineaments of spatter cones and scoria cones, with rootless flows forming by coagulation of spatter (e.g. Head and Wilson 1989). The lava flows produced can be expansive and inundate the land, developing lava tubes and interacting with surface water bodies as their path is channelled towards wet ground (Hamilton et al. 2010), potentially forming rootless vents tens of kilometres away from the fissure (Reynolds et al. 2015).

**Fig. 2:**
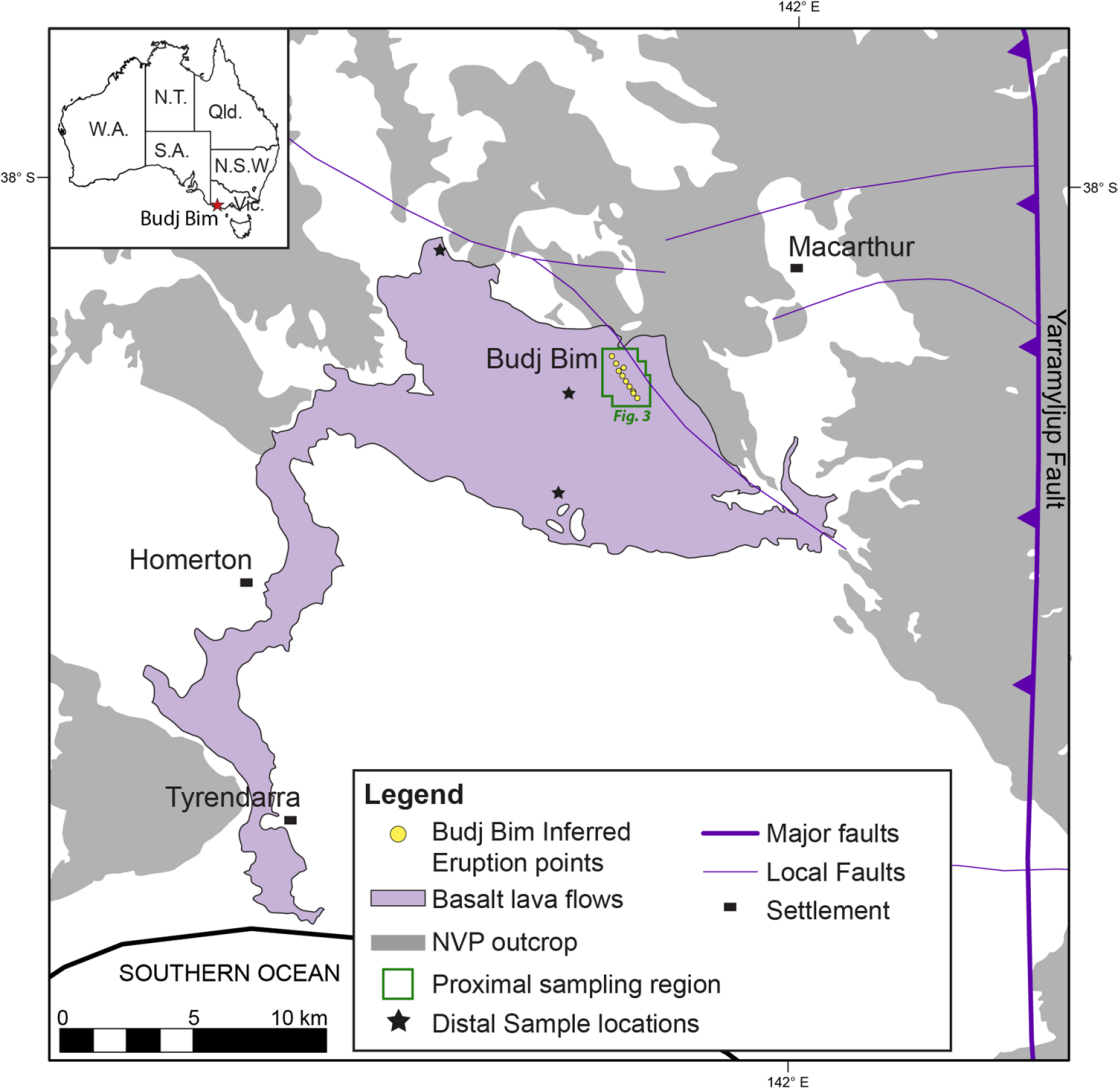
Map showing the regional volcanic deposits and geological structures surrounding the Budj Bim Volcanic Complex (BBVC) and distal sampling locations from this study. Geological boundaries are based on the Victoria State Government Earth Resources (GeoVic3) 250 k Geological Unit map and Eruption Points map. The green box indicates the main study area and proximal sampling region (see Fig. 3). See inset map for regional location

In continental monogenetic settings, eruptions can occur on short timescales and with short notice (Foote et al. 2022), potentially after long quiescence (Pallister et al. 2010). Understanding the mechanics of dyke ascent in these settings and the drivers of rapid transitions in explosive to effusive behaviour is important to accurately assess volcanic hazards. A common hindrance to progress in understanding the dynamics of volcanic fissure eruptions is that their deposits become quickly inaccessible and buried, with complex internal architecture created by the repeated growth and disruption of volcanic structures concealed by subsequent events (Holm 1987). The Budj Bim Volcanic Complex (BBVC, Fig. 1) is a rare example of an exposed volcanic fissure vent system in the NVP (Boyce 2013) and offers an important opportunity to address this issue. It resides within the Budj Bim National Park (formerly known as Mt Eccles National Park, co-managed by the Gunditjmara Traditional Owners and Parks Victoria) in the traditional Country of the Gunditjmara people in southeastern Australia (Fig. 2), 8 km west of the town of Macarthur in the Moyne Shire, southwest Victoria. The aim of this study is to reconstruct the eruptive history of the BBVC by combining new geological mapping, stratigraphic logging, petrography and geochemical analysis of erupted products with geomorphological constraints. We discuss our findings within a modern understanding of magma ascent and eruption dynamics to improve understanding of volcanic fissure eruptions and their impacts around the world.

## Geological and historical setting

The NVP is an active volcanic province (Cas et al. 2017) but there is no simple age pattern of eruptions across the NVP, and the main eruptive phase appears to have been an intermittent event between 4.5 Ma and 5 ka (Gray and McDougall 2009). The youngest dated eruptions in the NVP are those at Mount Schank and Mount Gambier, approximately 5000 years ago based on radiocarbon dating of charcoal (e.g. Blackburn et al. 1982). The cause of NVP volcanism is not well constrained, with relations to the breakup of Gondwana 90–55 Ma proposed (Veevers 1986) and trans-tensional decompression of hot and hydrated mantle (Price et al. 1997; Lesti et al. 2008; Jiang et al. 2016; Cas et al. 2017) as well as the effects of ‘edge driven convection’ at the step in the lithosphere beneath the edge of the continental crust (Demidjuk et al. 2007).

Lithospheric-scale structures have been suggested to control the geographical distribution of the volcanoes in the NVP (Boyce 2013; Van Otterloo et al. 2014; Cas et al. 2017; van den Hove et al. 2017a). The basement sequences underlying the BBVC are igneous and metasedimentary rocks from the Palaeozoic Kanmantoo Fold Belt rock successions, which are late Precambrian to Cambrian passive margin sequences deformed by the late Cambrian-Ordovician Delamerian Orogeny (Foster and Gleadow 1992). They are overlain by Lower Cretaceous to Eocene successions of the Otway Basin continental margin rift (e.g. Finlayson et al. 1996; Bryan et al. 1997). Major structures in the basin include Early Palaeozoic faults, such as the west-dipping listric Yarramyljup Fault which acted as a major border fault reactivated during Cretaceous rifting (Chukwu et al. 2025). It facilitated segmentation of the Cretaceous Otway Basin when Early Cretaceous normal (bounding half graben) and transfer faults of the northwest-southeast trending Ardonachie Trough (Vujovic et al. 2021) formed during the protracted break-up of Australia and Antarctica (Cas et al. 2017; Ziesch et al. 2017); these continued to accommodate spreading into the Eocene (Chukwu et al. 2025) with normal movement on growth faults. In the Tyrendarra embayment (near to which the BBVC lies) this involved secondary west-northwest faults (Fig. 2) dominating over the early east-northeast and east trending faults (Finlayson et al. 1996). A northwest trending strike-slip fault system developed during post-Oligocene faulting in the Otway Basin is associated with dextral trans-tensional tectonics (Miller et al. 2002). A shift to compressional tectonics occurred Late Eocene to present, due to Australia shifting north and impacting the Pacific and Eurasian plates (Rajabi et al. 2017).

The NVP volcanoes have erupted since the Pliocene and thus when the crust in Australia has been in a compressional tectonic setting (van den Hove et al. 2017a), so sill formation would be expected to be dominant over dyking (Menand et al. 2010; e.g. Magee et al. 2016). van den Hove et al. (2017a) suggest that an important facilitator to eruptions in the NVP will therefore be the cases where, rather than deflecting or stalling into sills, dykes ascending from depth will have intersected favourably oriented faults in the shallow crust. Many of the NVP volcanoes are close to major faults (Fig. 1; Boyce (2013)), and some scoria cones and maars in the province have a linear arrangement. Faults oriented nearly parallel to the maximum compressive stress (σ_1_) have been more often utilised by magmas compared to faults of other orientations (van den Hove et al. 2017a). Common eruptive products of the NVP are lava flow fields, lava plains, lava shields, scoria cones and maars, with magma compositions ranging from picrite to basaltic andesite (Cas et al. 2017). Phreatomagmatic activity in the south of the NVP is associated with the Otway Basin, where magma erupted through Tertiary sedimentary aquifers to produce maar volcanoes (Joyce 1975; Jordan et al. 2013; Boyce 2013). NVP eruptions in the north were dominantly magmatic as magma passed through Palaeozoic rock assemblages (Joyce 1975; Boyce 2013; Boyce et al. 2015).

**Fig. 3:**
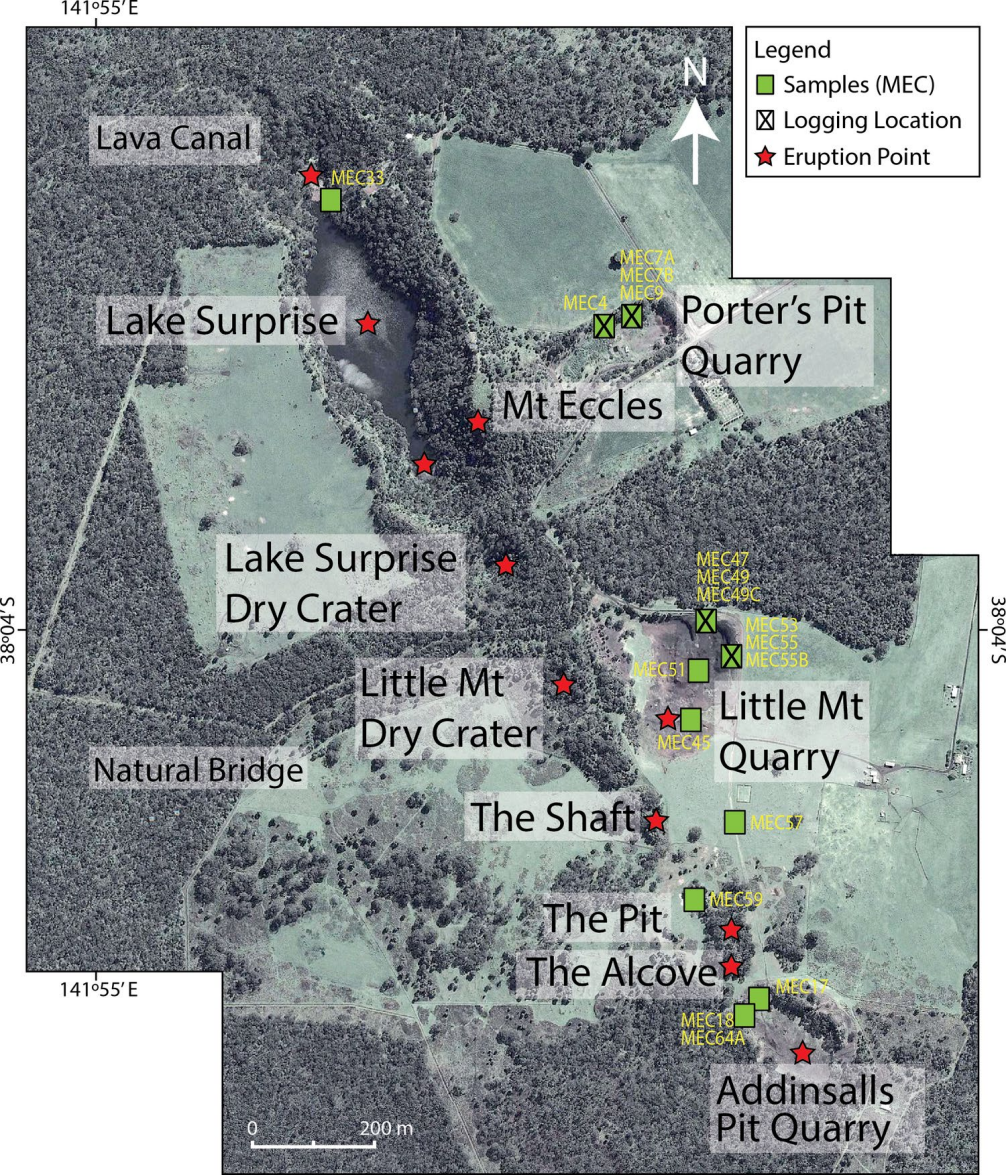
Locality photomap of the Budj Bim Volcanic Complex (BBVC). Google Earth imagery (2011 from CNES/Airbus) showing the major named volcanic features, inferred locations of volcanic eruption points and sampling localities (yellow text). The locations where generalised logs were completed are also shown

The BBVC is a multi-vent basaltic-trachybasaltic volcanic fissure complex with an extensive lava flow field, lava tube network and an elongate steep-sided open crater filled with a freshwater lake and aligned with several scoria and spatter cones (Ollier 1964a). A north-west south-east fault, mapped as part of the Delamerian Fold Belt (514 ± 3–490 ± 3 Ma; Foden et al. 2006), runs through the area and is parallel to but offset from the 2.2 km long BBVC array (Fig. 2). Ten inferred eruption points make up the main BBVC array (Fig. 3), though features such as Bald Hill shield edifice also lie on the same trend (GeoVIC 180; Boyce 2013), leading to up to twenty eruption points being proposed (Ollier and Joyce 1964). Such alignments of numerous spatter and scoria cones are relatively rare in the NVP (Bishop 2007; Lesti et al. 2008; van den Hove et al. 2017a), and the BBVC includes the only preserved elongate open crater with inferred eruption points in the NVP. Its main volcanological features are variably preserved, as quarrying, natural collapse, road building, farming, firebreak and transport infrastructure have modified or disturbed the landscape.

Budj Bim means ‘High Head’ in the Dhauwurd Wurrung language (https://youtu.be/akMqRFcWF2s, accessed 20th February 2026) and in the oral traditions was an ancestral being who transformed into the volcano. The Tyrendarra lava flow field (Fig. 2) is the most voluminous component of the BBVC and covers an area of approximately 140 km^2^. It extends ~20 km east to west and narrows to a ~2 km wide flow that has travelled over 30 km south to the Southern Ocean and reportedly up to 16 km offshore (Gill 1979; Grimes 1995). ^40^Ar/^39^Ar dating of the BBVC lavas suggests the eruption occurred 36.9 ka (± 3.1 ka, 95% confidence interval; Matchan et al. 2020), placing it within the context of early human occupation (Wilkie et al. 2020) when the eruption may have been witnessed, hence its preservation in oral traditions, and supporting postulations that the BBVC erupted at a similar time to nearby Mt Napier (Oostingh et al. 2017). A deep time story narrated by the Gunditjmara places them living in the Budj Bim Cultural Landscape for at least 32,000 years, with the continuation of cultural practice and oral transmission ensuring that the relationship between them and Budj Bim continues today (Wettenhall 2010). The lava flows have produced an extensive network of tubes whose caves have been explored and mapped since the 1960 s and 70 s (Ollier 1964a; Joyce 1976; Grimes 1995; Webb 2023). The Gunditjmara used the channels, weirs and dams created by these lava flows to trap, store and harvest short-finned eels (kooyang), providing a social base and economic prosperity for Gunditjmara society for 6000 years (McNiven et al. 2012; Brown 2024). The Budj Bim National Park obtained UNESCO World Heritage status in 2019, recognising its significant cultural heritage related to the development of one of the world’s oldest and most extensive examples of aquaculture (Wettenhall 2010).

## Methodology

### Volcanological mapping, stratigraphic logging and sampling

The volcanic deposits were mapped, sampled and stratigraphically logged during three field campaigns (two in 2012 and one in 2023). A DJI Mini 2 UAV equipped with a 12 megapixels camera was used to record spatial relationships and geomorphological features at higher elevation. Stratigraphic logging was completed at the Little Mount Quarry and Porter’s Pit Quarry (Fig. 3) where cuttings revealed excellent exposure of the volcaniclastic sequences; the landowners also cut new temporary trenches to facilitate our study. Basic rock characterisation was completed through outcrop inspection, and representative samples were collected for petrographic and geochemical analysis.

### Petrographic and image analysis methods

A set of representative polished thin sections (30 µm thick) were prepared for petrographic analysis, first using optical microscopy to assess the minerals present and their textures (see Supplementary Text) and using a blue-dyed resin to aid pore/vesicle identification. A sub-set were prepared for scanning electron microscopy (SEM) analysis by coating with a 15–20 nm thick carbon film to avoid charge accumulation. A ZEISS Gemini SEM 450 in the SEM Shared Research Facility (SRF) at the University of Liverpool was used to collect backscatter electron images (BSE) using a 1 nA beam current, 15 kV accelerating voltage and 9.3 mm working distance.

### Geochemical methods

Pyroclastic scoria and spatter samples and coherent lava samples were assessed for signs of alteration and/or weathering, and a sub-set of 25 samples was selected for whole-rock geochemical analysis, aiming for representation across the eruption sites and based on our new volcanic stratigraphy model (Table S1). This includes three archive samples from a previous study conducted by Monash University (Trowbridge 2003) selected for re-analysis so that the new data could be compared with previous trends. Major (25 samples) and trace (18 samples) elements were measured using X-ray fluorescence spectroscopy (XRF). Based on the whole-rock XRF results, a subset of six samples was selected for Sr-Nd-Pb isotope analysis. The detailed methodologies for major and trace-element whole-rock geochemistry and isotope analysis are described in Section A2 of the Supplementary Materials.

## Results

**Fig. 4:**
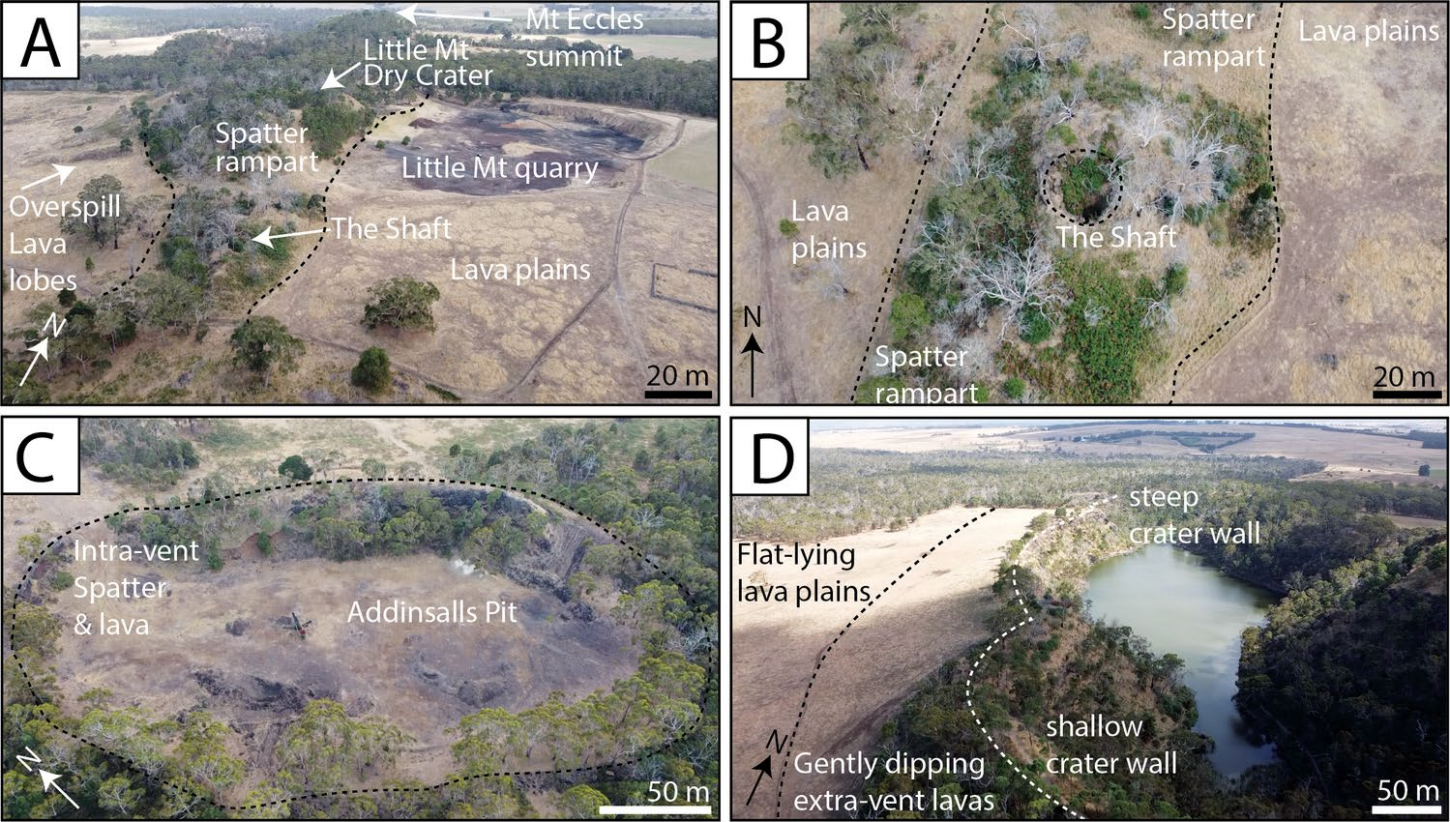
UAV photographs collected January 2023, showing some of the key volcanic structures of the BBVC (see Table S2 for GPS information). **A** The southern eruption points showing overspill lavas to the west, a linear spatter rampart and surrounding lava plains (indicative scale bar for the foreground). **B** ‘The Shaft’ with surrounding spatter rampart and lava plains (looking down). **C** The approximate outline of a heavily quarried spatter cone at Addinsalls Pit quarry. **D** Lake Surprise volcanic fissure crater, with its scalloped margin and surrounding lava plains (indicative scale bar for the foreground)

**Fig. 5:**
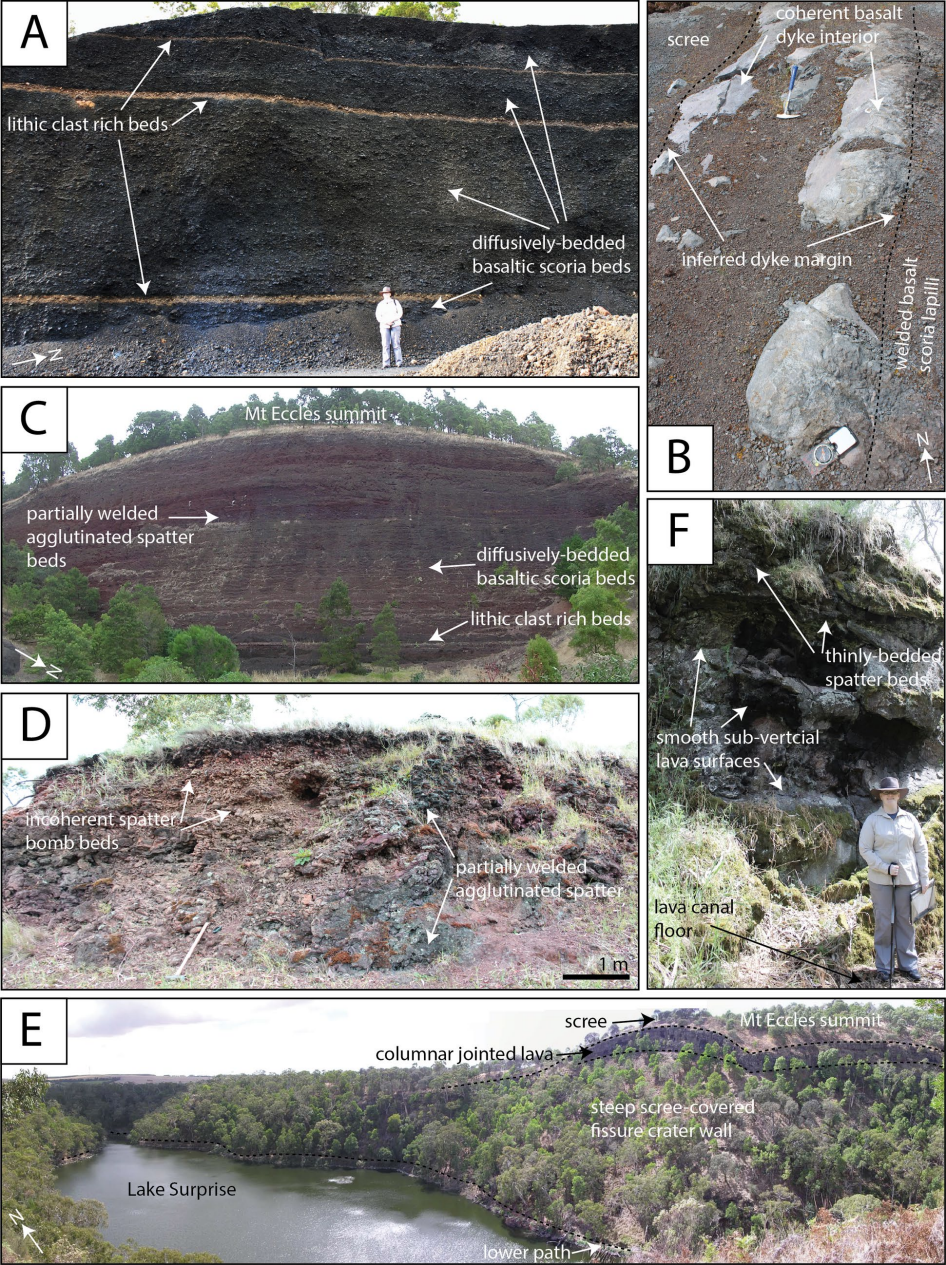
Photographs showing the internal architecture of key volcanic structures of the BBVC. **A** Scoria, spatter and lithic clast–rich beds exposed at the Little Mount Quarry. **B** Feeder dyke segment exposed within the Little Mount quarry floor (compass and hammer for scale). **C** Scoria and spatter deposits (partially welded) exposed in the southwest cutting at Porter’s Pit—the exposed face is ~100 m wide and ~45 m high. **D** Spatter mound and rampart exposed near The Pit and The Alcove. **E** View from the upper path, showing columnar-jointed lava flows near the summit of Mt Eccles (approx. 43 m above the lower path) and scree-covered steep fissure crater walls extending down to the lower path and Lake Surprise. **F** Thick basalt flow and lava levee at Natural Bridge—smooth surfaces suggest a lava channel has drained

The main sites which are the focus of this study are (1) Little Mount Quarry, located  ~200 m east of the ‘Little Mount Dry Crater’ (Figs. 4A and 5A,B), (2) Porter’s Pit Quarry, excavated into the flank of Mount Eccles (Figs. 4A and 5C), (3) The Shaft, The Pit and Addinsalls Pit Quarry (Figs. 4B,C and 5D) and (4) Lake Surprise volcanic fissure crater and lava flow fields (Figs. 4D and 5E,F). Their volcanic structures and deposits are first described (see Table S2 for geographic coordinates of key localities) followed by interpretations and additional insights inferred from cross-correlations between sites. Petrographic and geochemical results from stratigraphically constrained samples are then described.

### Volcanic structures and deposits

#### Little Mount Quarry

**Fig. 6:**
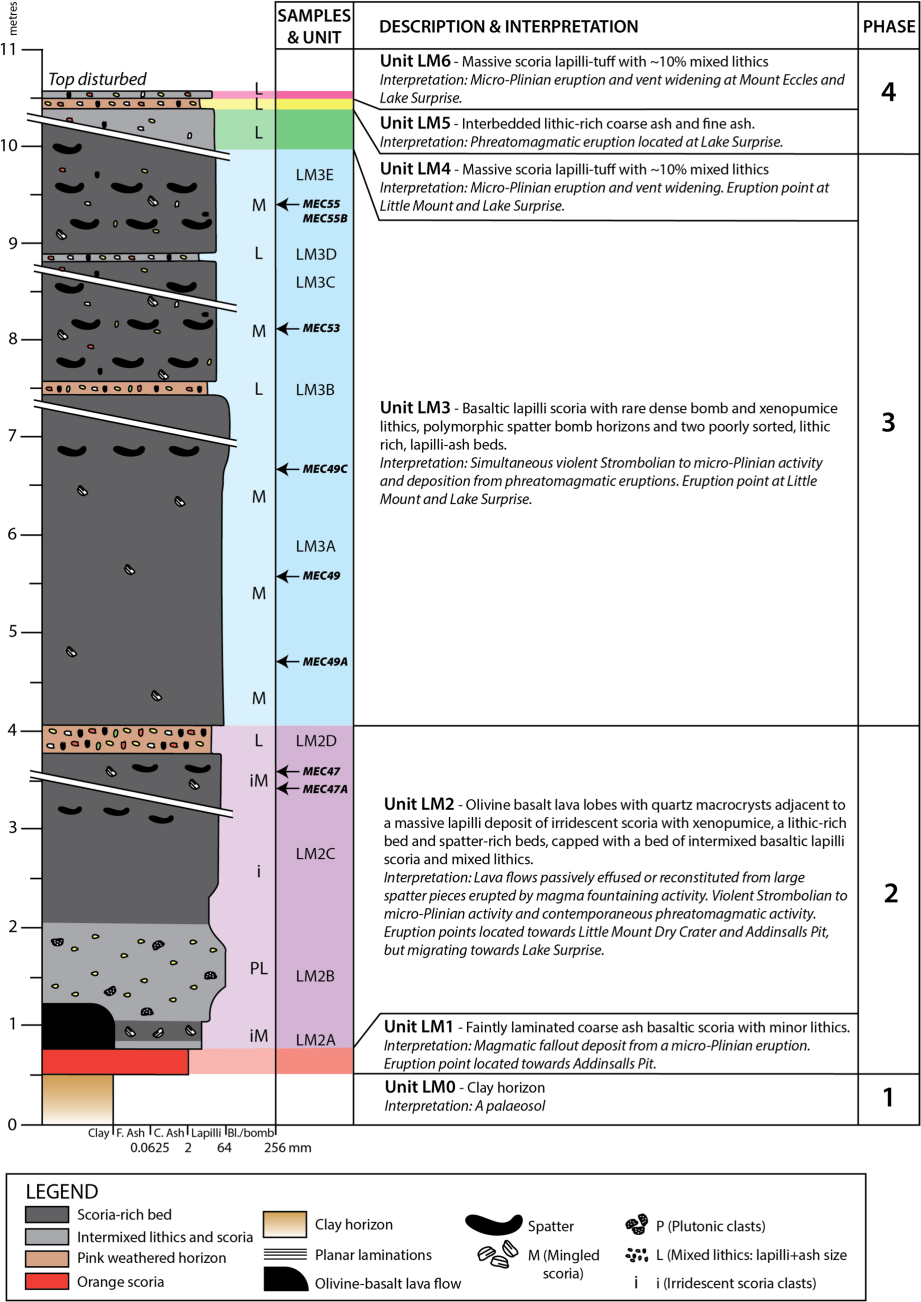
Composite log of deposits and interpreted eruptive phases recorded at Little Mount Quarry. The breaks in the log indicate five locations within the quarry where sequences were accessed

Stratigraphic logging and sampling were completed on excavated faces on the eastern and northern walls of a ~260 m long, ~200 m wide and ~10 m deep cut platform at Little Mt Quarry (Figs. 4A and 5A), with new 1–2 m deep trenches cut into the quarry floor to access deeper deposits (Figure S1). A composite log was produced based on descriptions and interpretations (Fig. 6 and Table 1).

**Table 1:** Little Mount Quarry stratigraphic unit descriptions, observations and interpretations of eruption processes and volcanic events associated with the Little Mount composite log (Fig. 6)

Unit	Sub- unit	Observations	Process interpretation	Interpreted event
Unit LM0	-	Red clay bed	Palaeosol formation	Reflecting a swampy palaeogeography
Unit LM1	-	~20 cm thick orange layer of faintly laminated coarse ash scoria (90%), scoria blocks (5%, ~ 5 mm) and red lithic clasts (5%). It has a sharp lower contact with the clay bed below. The scoria are orange stained (80%) and black (20%)	Magmatic fallout deposition	Micro-Plinian activity at a nearby eruption point. Palaeosurface clay bed is fragmented and mixes with tephra
Unit LM2	LM2A	The base of this 3-m thick sequence comprises an olivine basalt lava with quartz macrocrysts. Where the lava is not present, instead a massive black, iridescent lapilli scoria layer has a sharp contact with Unit LM1. The scoria are glassy and angular and there are occasional 1 cm size white foamy lithic clasts (mingled pumice)	Dominantly magmatic eruptions with fragmentation and co-production of lava flows. Occasional magma-water interactions cause explosions which excavate country rock fragments	Lava flows (LM2A) passively effused from Little Mount Dry Crater or reconstituted from large spatter pieces erupted by magma fountaining activity at the vent. Increasing explosivity over time with violent Strombolian to micro-Plinian activity building up Little Mount scoria cone (LM2A, LM2C) and contemporaneous phreatomagmatic activity from the Lake Surprise eruption point nearby (LM2B, LM2D)
	LM2B	~1 m thick layer of grey scoria (90%) and lithic clasts (10%) including angular plutonic lithic clasts (~4%) and beige clasts of probable sedimentary origin (6%). Diffuse contact with UNIT LM2A		
	LM2C	~1.5 m thick layer of black and iridescent basaltic lapilli scoria (98%, average size 12 mm, ranging from 5 to 20 mm) with rare 30 cm elongate basalt spatter bombs and occasional lapilli-sized quartz-rich lithic clasts and mingled scoria (2%). Diffuse contact with LM2B		
	LM2D	25 cm thick beige layer of intermixed basaltic lapilli scoria (85%) and mixed lithic clasts (15%) of dense basalt lithics, sandstone, mudstone and clay (1–20 mm). It has sharp upper and lower contacts		
Unit LM3	-	~6 m thick sequence comprising three well-sorted basaltic lapilli scoria layers and two distinctive interbedded ash and lithic-rich layers. Black scoria beds (3–14 m thick beds) include rare lithic clasts (2%), which are a mixture of dense basalt blocks and white crystalline lithic clasts. Spatter bomb-rich horizons are dispersed through the scoria beds, and polymorphic spatter bombs are up to 30 cm in length. The lithic clast–rich beds are poorly sorted, matrix supported and comprise basaltic lapilli scoria (70%) and lithic clasts (30%) with interstitial ash	Sustained deposition from magmatic eruptions, interspersed with pulses of magma-water interactions	Simultaneous violent Strombolian to micro-Plinian activity building up the Little Mount scoria cone and deposition from phreatomagmatic eruptions located at the Lake Surprise eruption point nearby
Unit LM4	-	Massive lapilli tuff which is rich in scoria lapilli and 10% lithic clasts. Lithic clasts are white lithic fragments and some dense basalt lithics (2 mm to 2 cm in size)	Magmatic eruptions with occasional vent-clearing blasts	Micro-Plinian activity building up the Little Mount scoria cone
Unit LM5	-	45 cm thick stratified orange/pink/brown bed that comprises lithic-rich coarse ash overlying an interbedded sequence of massive lithic clast–rich and massive fine ash	Sustained magma-water interaction	Phreatomagmatic eruptions located at the Lake Surprise eruption point nearby
Unit LM6	-	Massive lapilli tuff lithic clast–rich unit of lapilli scoria with dense basalt lapilli lithics 5–10 mm in size	Dominantly magmatic explosivity with occasional vent explosions blasting out country rock	Micro-Plinian activity, potentially related to the build-up of the Mount Eccles scoria cone

The base of the sequence is a red/orange clay (Unit LM0, Figure S1A) and six volcanic units overlie this. Unit LM1 is a red, faintly laminated coarse ash (Figure S1A, B). Unit LM2 is divided into four sub-units, starting with an olivine basalt lava which has lobate architecture and contains rare quartz macrocrysts (Unit LM2A, Figure S1B). Where the lava is not present, instead a thin grey bed of lithic clast–rich material abruptly contacts a massive black, iridescent lapilli scoria layer (Unit LM2A, Figure S1A). It contains occasional quartz-rich vesicular lithic clasts which are mingled with basalt scoria. The deposits transition into basalt scoria beds which contain up to 15% lithic clasts including sandstone, siltstone, clay, dense basalt, quartz-rich mingled basalt scoria and plutonic clasts including pyroxenite (Unit LM2B, Figure S1A, B), a massive lapilli scoria (Unit LM2C, Figure S1C) and a lithic clast–rich bed with pink interstitial ash (LM2D, Figure S1C). Unit LM3 comprises 1.5–3 m thick beds of well-sorted basaltic lapilli scoria with two 10–30 cm thick ash and lithic clast–rich interbeds (Figure S1C, D, E). The scoria beds have rare dense mafic and white crystalline lithic clasts (2%), whereas the lithic-rich beds include mixed sedimentary and igneous lithics (like those described in Unit LM2). Unit LM4 is a 0.5 m thick unit of massive lapilli tuff that is rich in scoria (Figure S1F). Following this, Unit LM5 is an interbedded sequence of massive lithic clast–rich and massive fine ash overlain by a lithic clast–rich coarse ash (Figure S1F). Unit LM6 tops the sequence with a massive lapilli tuff and clast-rich lapilli scoria with dense basalt lithic clasts (Figure S1F). A layer of fine-grained clasts which is structureless and contains some minor lithic fragments tops the sequence and is interpreted to be reworked (Figure S1F).

On the quarry floor, linear structures of dense, coherent basalt with sharp sub-vertical contacts with welded basaltic lapilli scoria were exposed (Fig. 5B and Figure S2). Some of these segments are oriented approximately northwest-southeast (parallel to the fissure trend) and some are north-northeast (~60° offset from the fissure trend).

#### Porter’s Pit

**Fig. 7:**
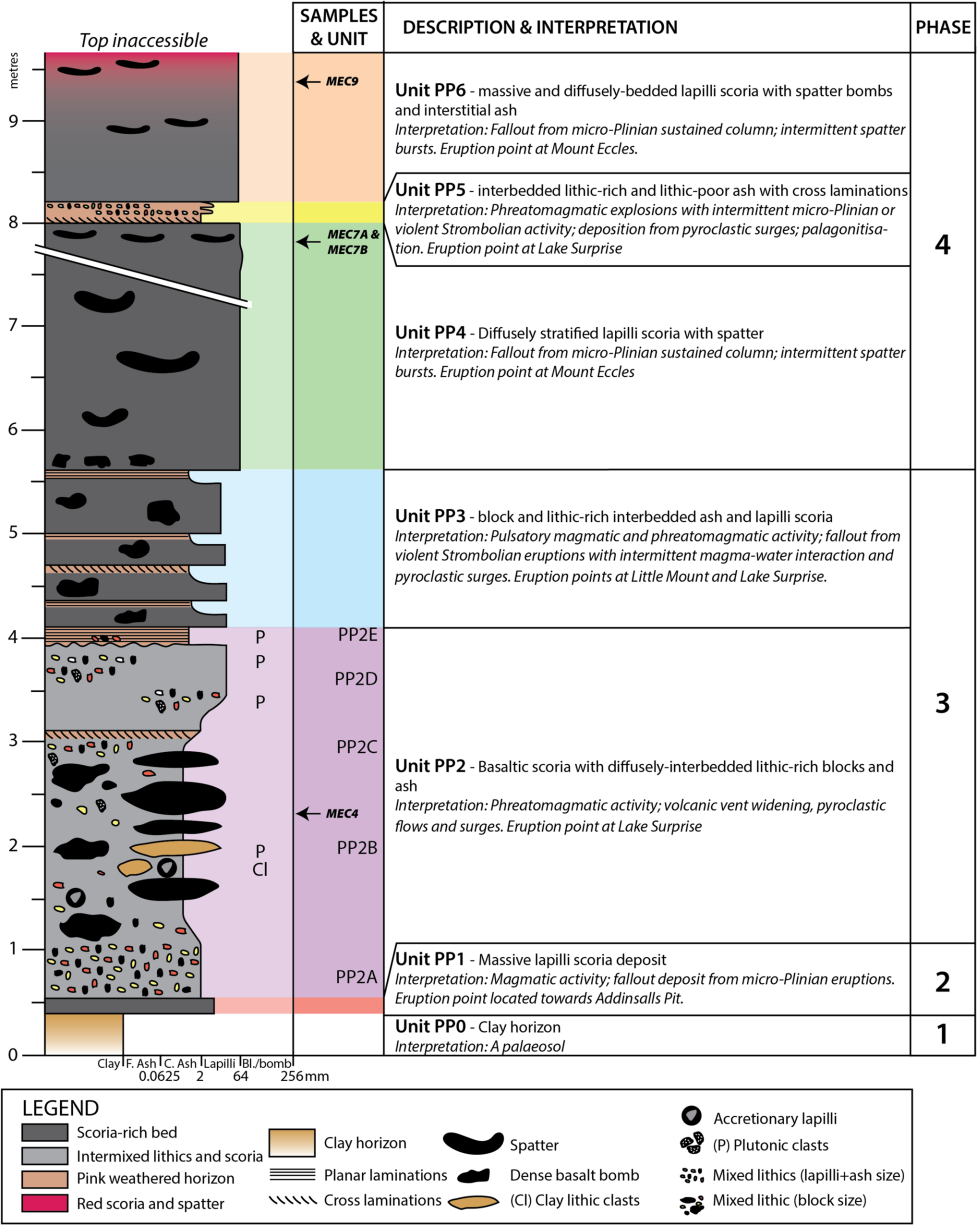
Composite log of deposits exposed by quarry cuts at the Porter’s Pit Quarry and interpreted eruptive phases. The break in the log indicates two nearby locations within the quarry where sequences were accessed

The summit of Mount Eccles marks the highest point of elevation in the BBVC (160 m above sea level) and is adjacent to the most southerly eruption point at Lake Surprise (Fig. 3). We suggest this was the main volcanic cone of the BBVC based on its large size and topography. The Porter’s Pit quarry excavated into its northeastern flank with three cuttings providing excellent exposure and amphitheatre-style access to its internal architecture and surrounding deposits. The southwest cutting (Fig. 5C, S3) is approximately parallel to the BBVC trend and is the tallest exposure at the locality (~45 m high and ~120 m wide). It has been a dominant feature of the site for decades (Figure S4). Two perpendicular ~230 m long cuttings in the northwest and southeast of the site expose deeper deposits (Figure S3). Stratigraphic logging and sampling were completed on the northwest cutting to create a composite log based on descriptions and interpretations (Fig. 7 and Table 2).

**Table 2:** Porter’s Pit stratigraphic unit descriptions, observations and interpretations of eruption processes and volcanic events associated with the Porter’s Pit composite log (Fig. 7)

Unit	Sub- unit	Observations	Process interpretation	Interpreted volcanic event
Unit PP0	-	Lower layer is a beige structureless, very well sorted, mud dominated clay unit with 5% silt, overlain by a grey/beige clastic bed with an orange top. This has an undulous upper contact with a 2 cm thick yellow-orange clay unit that is very well sorted and finely laminated	Palaeosol formation	Reflecting a swampy palaeogeography
Unit PP1	-	10 cm thick, black massive lapilli scoria unit comprising lapilli scoria (99%, ~3 mm) and minor free crystals (< 1%, ~ 1 mm). Sharp lower contact with the underlying clay unit	Fallout from magmatic explosivity	Violent Strombolian to micro-Plinian activity at a nearby eruption point
Unit PP2	PP2A	~30 cm thick unstructured, poorly sorted and lithic clast–rich (80%) deposit with minor basalt scoria lapilli. Lithic clasts are lapilli and ash sized fragments of mixed composition—limestone (beige), dense basalt (black) and sandstone (red). Sharp contact with Unit PP1 below. Fines upwards, grading into Unit PP2B	Gas-rich/magma-poor eruptions and/or magma-water interactions. Discrete pyroclastic surges and alternating pulses of en-masse and localised deposition with traction. Erosive pyroclastic flows were periodically carving into recent deposits and remobilising material. Fine ash settled out from suspension between flows. Presence of lithic blocks and bombs suggests location is within the ballistic zone	Phreatomagmatic activity originating from a Lake Surprise eruption point. Volcanic vent opening, with explosivity increased due to magma interaction with an aquifer
	PP2B	2 m thick poorly sorted, matrix supported, and occasionally laminated deposit dominated by basaltic lapilli scoria. Dispersed lapilli and block-sized lithic clasts of dense angular porphyritic basalt lapilli, clay lithic clasts (beige/yellow), limestone, rare angular plutonic lithics (possibly pyroxenite) and rare accretionary lapilli. Occasional discontinuous bed-parallel normally graded lenses. Occasional large elongate blocks and bombs of angular, vesicular basalt (25–40 cm long) and very rare (< 1%) large elongate clay blocks (3–20 cm long). Matrix is a pale brown lithic clast–rich ash. Grades into Unit PP2C		
	PP2C	~20 cm thick red coarse ash layer comprising black and red grains (30:70 ratio) and cross laminations. < 5% basaltic scoria. Sharp upper contact with Unit PP2D		
	PP2D	~1 m thick dark grey bed, moderately well sorted basalt scoria and lithic clast–rich lapilli comprising dense basalt, limestone and rare plutonic lithic clasts and large (30 cm) dense basalt bombs		
	PP2E	30 cm thick brown coarse ash bed with < 20% basaltic scoria lapilli. Finely laminated, poorly sorted and comprises a diverse range country rock lithic clast types (white, black, grey, red) and including rare plutonic lithic clasts. Imbricated lapilli-sized lithic clasts (1 cm, dense basalt) at its base. The contact with Unit PP2D is undulous and interpreted to be erosional		
Unit PP3	-	~1.5 m thick dark grey sequence of lapilli scoria beds with interbedded block-rich layers and brown lithic clast–rich coarse ash. Ash beds are 5–10 cm thick, are finely laminated, poorly sorted and occasionally cross bedded. They comprise a diverse range country rock lithic clasts (dense basalt, orange sandstone, clay, siltstone). Rare white vesicular and white crystalline clasts within basaltic lapilli scoria. The scoria beds are 10–50 cm thick and are normally graded with occasional dense basalt bombs. Each scoria bed is overlain by ash with sharp contacts	Intermittent fallout from magmatic eruptions with periodic magma-water interactions and the generation of pyroclastic surges	Violent Strombolian eruptions and pulsed phreatomagmatic explosivity originating from a Lake Surprise eruption point
Unit PP4	-	~2.5 m thick bed of black diffusely stratified and well-sorted lapilli scoria. Occasional (1%) orange-stained vesicular basalt. At the base of the bed there are a few dense basalt bombs, and moving up through the sequence there are occasional spatter clasts. It has a sharp contact with the underlying Unit PP3	Magmatic explosivity with gentle pulsing and occasional ballistic spatter bombs	Micro-Plinian activity, starting to build the Mount Eccles scoria cone; intermittent spatter bursts within sustained micro-Plinian column
Unit PP5	-	20 cm thick pale orange layer of interbedded lithic clast–rich and lithic clast-poor ash with cross-laminations. Very low juvenile component (~3%). Thicker beds (~70 cm) in the southwestern cutting where it comprises several layers of ash separated by thin beds of scoria	Intermittent gas-rich/magma-poor eruptions erode out country-rock fragments, generation of pyroclastic surges and magmatic explosions	Phreatomagmatic explosions with intermittent micro-Plinian or violent Strombolian activity; wet ash has been palagonitised
Unit PP6	-	Massive and diffusely bedded lapilli scoria beds with spatter bombs and interstitial ash. The sequences changes colour from black to maroon, purple and red up sequence and some beds are welded in places	Magmatic explosivity	Fallout from micro-Plinian sustained column, building up the Mount Eccles scoria cone; intermittent spatter bursts

The base of the sequence (Unit PP0) comprises a beige structureless, very well sorted, mud-dominated clay unit with 5% silt, overlain by a grey/beige clastic bed with an orange top (Figure S3A). It has an undulous upper contact with a 2 cm thick lens of a yellow-orange-beige clay unit that is very well sorted with horizontal laminations. The first volcanic deposits are massive lapilli scoria (Unit PP1, Figure S3A). The following sequence is a basaltic lapilli scoria with diffusely interbedded lithic clast–rich blocks and ash (Unit PP2), divided into five sub-units (PP2A-E, Figure S3B). The lithic components comprise dense basalt, limestone, clay, siltstone and sandstone. Plutonic lithic clasts and accretionary lapilli also occur but are rare. The third group of deposits is lapilli scoria beds with interbedded block-rich layers and brown lithic clast–rich coarse ash (Unit PP3, Figure S3B). The subsequent deposition was of a diffusely stratified and well-sorted lapilli scoria deposit (Unit PP4, Figure S3C). Unit PP5 begins the return to deposition of interbedded lithic clast–rich and lithic clast-poor ash with cross-laminations (Figure S3D,F). Thin scoria-rich beds are locally interbedded but are only observed in the southwest cutting where the ash-rich beds are thickest (Figure S3C,F). The final deposits logged at Porter’s Pit are massive and diffusely bedded lapilli scoria beds with spatter bombs and interstitial ash (Unit PP6, Figure S3E,F).

#### Spatter ramparts and spatter cones

A linear ridge of elevated ground runs from the southern tip of the Lake Surprise Dry Crater south-southeast to a wooded area between Little Mount and The Shaft (Fig. 4A). It comprises dense, agglutinated basalt spatter beds which are oxidised red (e.g. Figure 5D). The ridge transitions southward into a lower-profile linear section including three inferred eruption points, locally known as ‘The Shaft’, ‘The Pit’ and ‘The Alcove’ (Fig. 3). These small, 10–30 m high spatter cones have open craters and cogenetic lava flows (Tunjc 1988). They comprise agglutinated vesiculated basalt spatter bombs which have highly irregular margins and are polymorphic. There are occasional metre-scale lenses of dense basalt. The spatter bombs are oxidised to a red/orange colour. The Shaft (Fig. 4B) is reported to be at least 30 m deep (Ollier 1964a; Rosengren 1994). East of the Little Mount Dry Crater, a 1.5 m thick bed rich in ~2–10 cm diameter red, rounded clasts crop out, and when cut open, these reveal a lithic core (Figure S5).

Addinsalls Pit Quarry is an oval structure located in the most southerly point of the BBVC (Fig. 4C). Its lowest exposed unit is a coherent vesicular basalt lava flow (Figure S5D) (Trowbridge 2003) overlain by scoria, followed by red-oxidised spatter, which is variably agglutinated, and an inclined basalt lava flow (approximately one metre thick) (see Figure S6). Quarrying efforts perhaps would have focused on the removal of scoria, and the size and shape of the site at Addinsalls Pit are typical of a scoria cone. ‘Huggins Mount’ is mentioned on schematic sketches of the BBVC from the National Parks guidebook (see Supplementary Materials Figure S1), where it looks as though it would be depicting a scoria cone, although this is now broadly flat-lying land, perhaps excavated by quarrying.

#### The ‘Lake Surprise’ volcanic fissure crater and lava flow fields

The ‘Lake Surprise’ volcanic fissure crater (Fig. 4D) is an approximately 900 m long and up to 300 m wide (Fig. 5E) elongate water-filled crater in the north of the BBVC. The fissure is approximately symmetrical along its centre line and scalloped by three rounded features, interpreted to be three coalesced craters from three eruption points (Fig. 3). The northern eruption point sits at 130 m above sea level, and the lake water depth is estimated to be ~11.5 m (Dharmarathna 2022). The fissure margin is best viewed from a flat, laterally continuous pathway along the lake margin and a path along the crater rim, each providing an amphitheatre-style view of the crater’s internal architecture exposed due to natural collapse (Fig. 5E). The elongate margin dips steeply into the crater at its centre but is more shallowly dipped towards its northern and southerly tips. The distance from the lake margin path to the crater rim increases towards the south-southeastern end of Lake Surprise, where the crater truncates the summit of ‘Mount Eccles’ (Fig. 5E). Directly to its south is the ‘Lake Surprise Dry Crater’, which is a vegetated and inaccessible isolate circular crater inferred to be an additional eruption point (Fig. 3).

The fissure margin is delineated by a break of slope from variably shallow and steeply dipping internal walls to gently outward dipping lavas which transition into flat-lying lava plains (Fig. 4D). The internal walls comprise alternating horizons of 'a'ā and pahoehoe lava and agglutinated spatter bomb horizons, with at least 10 separate lava flows identified based on cooling joint patterns (Figures S8, S9, and S10). These lavas are largely inaccessible due to the vertical crater wall and are partially collapsed. Cooling joint patterns mark the boundaries between successive 'a'ā and pahoehoe flows, and these suggest the thickest individual flow units within the crater reached a maximum of ~5 m, with minor flow units of agglutinated spatter being tens of cm thick. The flows are laterally irregular and sometimes discontinuous, probably reflecting lava lobes that flowed perpendicular to and away from the crater wall; it is often difficult to trace individual flows over a distance greater than 100 m in the fissure crater wall. Occasionally, the crater wall faces were observed to be smooth and very steep-sided and composed of a coherent basalt lava, with occasional linear cm-scale ‘drip’ features oriented into the crater (Figure S10). Columnar jointed lavas arch and drape the geomorphological landforms in their upper levels (Fig. 5E). The northern tip of the Lake Surprise volcanic fissure is marked by a breach at Lava Canal (Fig. 3) where thick lava sequences are abundant (Fig. 5F).

### Cross-correlations and interpretations

Units LM0 and PP0 are interpreted as clay-rich soil formed in a swampy environment similar to conditions when Aboriginal people developed fishtraps (McNiven et al. 2012). The observation of tumuli in the lava field at Lake Condah and Lake Gorrie is consistent with the palaeosurface of the BBVC eruptions being wet ground. Given the proximity of the quarries to one another, it is likely that deposits from a single eruption point are recorded at both sites. Several phases of eruption are thus interpreted based on the logged sequences, exposures and cross-correlations between localities. The brown colour of the ash throughout the area is interpreted to be due to hydration and palagonitisation of wet volcanic glass shards (Stroncik and Schmincke 2002).

#### Interpretations of deposits at Little Mount

LM1 is interpreted to be part of a widely dispersed fallout deposit from a micro-Plinian eruption which formed a nearby scoria cone (Giordano et al. 2024). We adopt the term micro-Plinian, proposed by Francis et al. (1990), which redefines sustained scoria-forming eruptions (historically termed ‘Strombolian’ by e.g. Walker (1973)) as distinct from Strombolian *sensu stricto* eruptions, which produce intermittent spatter-sprays and are typical of Stromboli volcano (Giordano et al. 2024). Micro-Plinian eruptions are thus defined as relatively small, sustained eruptions involving continuous discharge from a buoyant, convective eruption plume, whereas Strombolian eruptions are transient explosive ballistic sprays with characteristic discrete, short-lived and time-spaced events rather than a sustained convective column (Giordano et al. 2024). Micro-Plinian events produce typically massive deposits with minimal stratification, scoria cones near the eruption point and widely dispersed scoria fallout sheets.

The clastic sequences of LM2 are interpreted as fallout from violent Strombolian to micro-Plinian activity (Giordano et al. 2024), with occasional intense explosions, perhaps phreatomagmatic, eroding out country rock fragments (Kósik et al. 2016). Strombolian eruptions produce spatter and scoria, building small spatter cones or ramparts immediately near the eruption point but with a very minor amount of ash (Giordano et al. 2024). We interpret the lava at the base of LM2 to be either passively effused or a rootless flow (Head and Wilson 1989) reconstituted from large spatter pieces erupted by magma fountaining activity, perhaps at the Little Mount Dry crater or another nearby spatter cone.

Violent Strombolian to micro-Plinian activity is interpreted to have continued with the deposition of Unit LM3 but transitioned to be unsteady, interspersed with intermittent magma-water interactions. Some schematic representations of the BBVC show a hill adjacent to, but offset from, the fissure trend (Figure S7) and named Little Mount (now the location of the Little Mount Quarry) (Grimes 1995; Trowbridge 2003). Our observations support the interpretation of a scoria cone located ~200 m east of the Little Mount Dry Crater, making this the 11th eruption point of the BBVC. We suggest the dyke segments we identified were part of its feeder system, which intruded younger (NW–SE) as well as older (NNE) fault segments (van den Hove et al. 2017b). The Little Mount scoria cone produced the juvenile basaltic scoria, but we suggest an alternative eruption point produced occasional intense phreatomagmatic explosions, likely involving an aquifer, leading to the expulsion of lithic clasts. The discrete occurrence of lithic clasts within relatively thin beds suggests the location of magma-water interaction was remote from Little Mount; a strong candidate for the source location is the Lake Surprise fissure crater, suggesting simultaneous eruptions along the BBVC having very different eruption styles. Similar vent-shifting events and concurrent/alternating magmatic/phreatomagmatic eruptions with contrasting styles have been previously described (e.g. Kósik et al. 2016; Foote et al. 2022), further demonstrating complexity within small-volume volcanic events and their hazards. The LM4 deposits indicate continued micro-Plinian activity, but deposition of Unit LM5 marks the return to phreatomagmatism, characterised by a period of sustained magma-water interaction with an aquifer, again potentially linked to the active vent switching to be at Lake Surprise. LM6 indicates violent Strombolian to micro-Plinian activity from a remote eruption point, with occasional explosions blasting out country rock fragments from there.

#### Interpretations of deposits at Porter’s Pit

Unit PP1 is interpreted as part of a widely dispersed fallout deposit from a micro-Plinian eruption which formed a nearby scoria cone. Units PP1 and LM1 have similar composition and size, suggesting they derive from the same event.

The high proportion of coarse ash and angular lapilli lithic clasts subsequently deposited in unit PP2 can be attributed to a significant phase of vent opening via country-rock erosion and gas-rich/magma-poor eruptions, with possible magma-water interactions due to interaction with an aquifer (Van Otterloo et al. 2013). The combination of massive, structureless deposits and occasional lithic clast–rich lenses suggests the generation of discrete, sometimes erosional, pyroclastic surges (Kilgour et al. 2019). Cross-bedding suggests traction at the base of pyroclastic surges. Poorly sorted deposits suggest pulses of en-masse deposition from column collapse due to close proximity to the eruption point. The presence of lithic blocks and bombs also suggests the eruption point for these deposits was very close and that this site was in the ballistic curtain. The lithic clast types from PP2 are also present in LM2, but they are smaller and less abundant in LM2 than in PP2. We suggest these deposits indicate the vent has shifted to the Lake Surprise area, with the ascending magma interacting explosively with an aquifer (Kósik et al. 2016; Foote et al. 2022).

Unit PP3 indicates intermittent fallout from violent Strombolian eruptions and pulsed phreatomagmatic explosivity from either a single or multiple eruption points, again evidencing rapid and contemporaneous transitions in eruption style and location (Kósik et al. 2016; Foote et al. 2022). The deposits of LM3 and PP3 are both interpreted to have formed as fallout from violent Strombolian eruptions with intermittent phreatomagmatism. However, as the magmatic LM3 deposits are much thicker than the PP3 deposits, this suggests magmatism from the Little Mount scoria cone occurred alongside phreatomagmatism at a separate vent close to Porter’s Pit, located at Lake Surprise.

PP4 is interpreted to be fallout from more micro-Plinian explosivity with intermittent spatter bursts. We suggest magmatic activity dominated along the fissure at this time, starting to build the Mount Eccles scoria cone, and magma-water interaction was limited. PP5 is interpreted as the return to phreatomagmatism and the generation of pyroclastic surges, due to magma interaction with an aquifer and temporary shut-down of magmatic explosivity as the conduit was destabilised at Lake Surprise (Foote et al. 2022). The thin and locally interbedded scoria-rich beds suggest distal and intermittent violent Strombolian or micro-Plinian activity was ongoing from separate vents. PP6 represents a major magmatic phase in the eruption which built up the Mount Eccles scoria cone due to fallout from a sustained micro-Plinian column and intermittent spatter bursts as the eruption conduit was fully established (Foote et al. 2022).

#### Interpretations of spatter ramparts and spatter cones

The morphology of the elevated ridge running from Little Mount Dry Crater to the line of spatter cones that extend to Addinsalls Pit strongly resembles a spatter rampart developed during magma fountaining along a fissure (Giordano et al. 2024). Alternative models have suggested these were adventitious (rootless) vents created by lava (Ollier 1964a). However, the spherical clasts observed within the spatter rampart are interpreted to be armoured lapilli and armoured bombs, consisting of a lithic or scoria core mantled by juvenile basalt. These clasts are dense and large, and they would land close to the vent. They represent previously erupted scoria or lithic clasts re-incorporated into magma fountains, whereby the collapse of deposits around the crater moved juvenile clasts back into the vent, accreting a new layer of magma within a fluidised melt spray (e.g. Gernon et al. 2012); however, further detailed field observations and geochemical analysis would be needed to explore alternative interpretations as well (e.g. Murcia et al. 2015). No country rock lithic clasts were observed in the deposits, which would be expected if these were rootless cones (Reynolds et al. 2015). Overall, the evidence supports the spatter rampart and spatter cones being fed by an erupting dyke. We suggest that flow quickly focused along the fissure, and large spatter pieces fell back around the crater area, building up the spatter cones (Holm 1987). Some of the spatter likely became reconstituted as lava that flowed away from the eruption point, as was the case during the 2014–2015 Bárðarbunga-Holuhraun fissure eruption in Iceland (Pedersen et al. 2017). We suggest it is this process which explains the occurrence of lava at the base of the sequences in the Little Mount trenches (Unit LM2), and these may be rootless flows (Head and Wilson 1989).

Our interpretation of the deposits at Addinsalls Pit is that they record early violent Strombolian or micro-Plinian activity in this area (Giordano et al. 2024); however, detailed logging and sampling in this area would be needed to confirm this. This activity could be responsible for the deposition of LM1 and PP1 through a widely dispersed lapilli scoria fallout. The inclined lava flow at the edge of Addinsalls Pit post-dates the formation of the spatter layers and, although its eruption point location is unknown, it is likely to have originated as a highly agglutinated clastogenic lava from magma fountaining, likely linked to the spatter cones and rampart.

#### Interpretations of deposits at Lake Surprise

We interpret lavas exposed within the Lake Surprise elongate crater to be in situ agglutinate that laterally transformed into clastogenic lava, originating from large spatter pieces erupted due to magma fountaining within a lava lake accumulated to fill the length of an elongate, steep-walled crater (following Giordano et al. (2024)). Some of these lavas overspilled the crater, whilst others flowed back into the crater. The columnar-jointed lavas at the highest levels are most likely densely welded agglutinated spatter layers resulting from magma fountaining fallout from the lava lake onto high topography (Giordano et al. 2024). The breach at Lava Canal is where the lava lake fed a substantial lava flow field known locally and historically as the ‘stony rises’ (e.g. Fraser et al. 2025) due to the very irregular surface morphology of rises and depressions (Fig. 2). The lava flow field includes substantial lava caves and open channels (see Supplementary Materials, Figure S10, and Grimes (2002)) that have been mapped across the northwest and west of Lake Surprise and to the west of Little Mount (Natural Bridge, Figs. 3 and 5B). Tumuli have also been reported in the Lake Condah and Lake Gorrie swamplands further afield (Trowbridge 2003). They are very similar to those associated with the nearby lavas from Mt Napier (Ollier 1964b). The > 3 m-thick lava flows, which can be traced > 30 km to the sea and beyond (Fig. 2), indicate a sustained high-effusive phase in the eruption (Walker 1973).

Our interpretation is a lava lake within the fissure fed substantial lava outpourings, which agrees with previous studies (Edney 1987; Tunjc 1988; Trowbridge 2003). The maximum level the Lake Surprise fissure lava lake could have reached must be the lowest topographic level on the elongate open fissure crater wall at any time during the eruption because lava overspill would occur from the lowest point on the elongate crater wall. The Budj Bim lava lake at Lake Surprise and surrounding lava field would have looked similar to the 2014–2015 Bárðarbunga-Holuhraun eruption in Iceland, which fed > 58 km^2^ of basaltic lava flows from a ~500 m long section of a 1.8 km long fissure (Pedersen et al. 2017).

### Petrology and crystal textures

**Fig. 8:**
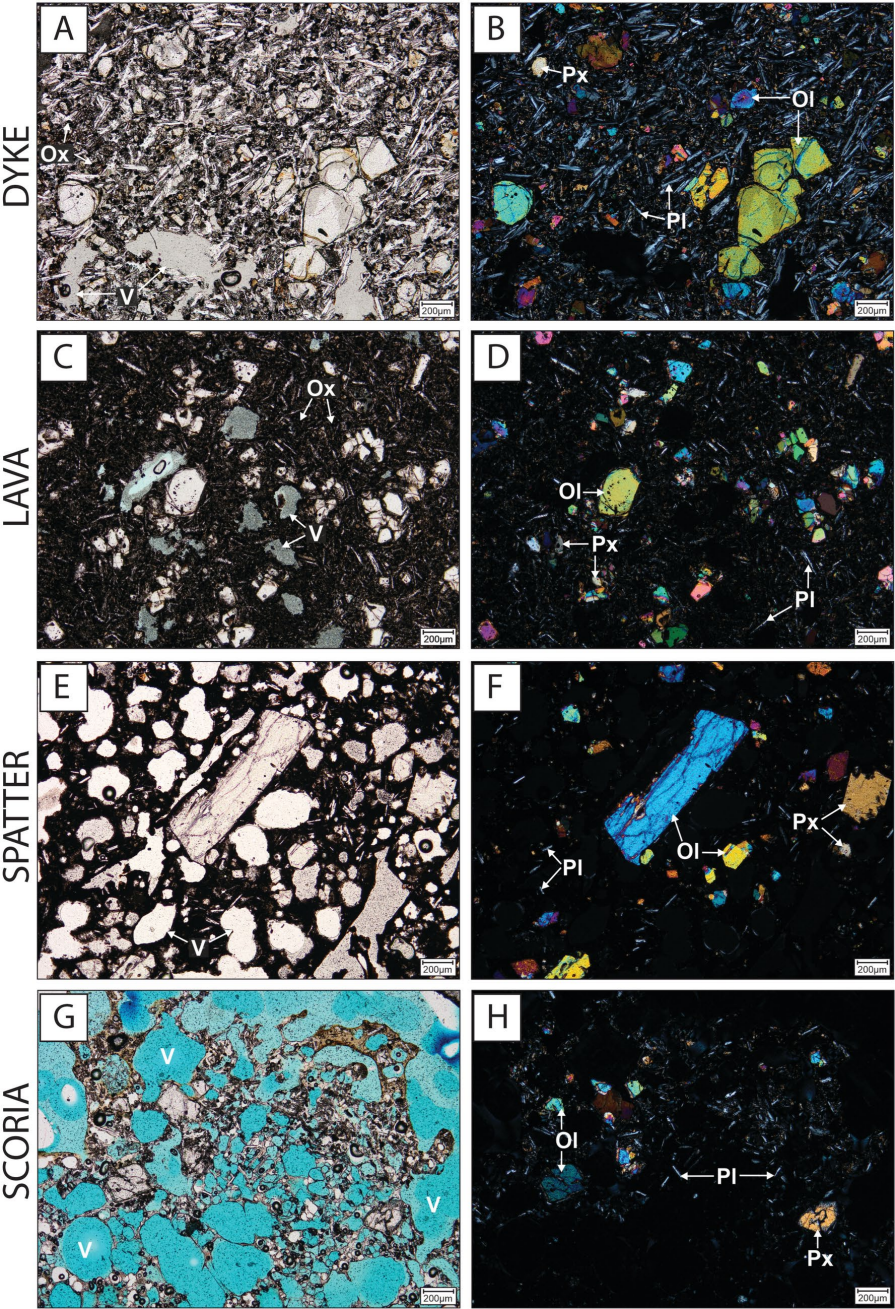
Representative photomicrographs showing the petrography and textures of representative BBVC samples. Coherent rock (dyke) sample from Little Mount (MEC45) showing porphyritic texture with olivine and clinopyroxene macrocrysts in an aphanitic/glassy groundmass and trachytic texture expressed by plagioclase microlites, viewed in PPL (**A**) and XPL (**B**). Lava from Addinsalls Pit (MEC64A) viewed in PPL (**C**) and XPL (**D**), showing euhedral olivine phenocrysts with melt inclusions. Spatter deposits at Porter’s Pit (MEC7B) showing vesicular texture, viewed in PPL (**E**) and XPL (**F**). Mingled scoria at Little Mount Quarry (MEC49C), showing blue-dyed vesicles and colourless/black glassy texture with rare microphenocrysts of olivine, plagioclase and clinopyroxene, viewed in PPL (**G**) and XPL (**H**). Ol—olivine, Pl—plagioclase, Px—clinopyroxene, Ox—oxide, V—vesicles. Blue-dyed resin is used in MEC64A (**C**) and MEC49C (**G**)

A suite of juvenile samples comprising scoria, spatter, lava and dyke material from across the BBVC was selected for petrographic study (Table S1). They are variably porphyritic and have macrocrysts (up to 2.5 mm in diameter) of olivine, plagioclase and clinopyroxene ± Fe-Ti oxides and quartz in order of decreasing abundance (Fig. 8). Vesicularity of the samples is variable, with scoria having the highest vesicularity (50–80% vesicles), whereas dyke, spatter and lava samples have lower vesicularity (10–40% vesicles). Groundmass textures are variably glassy or microcrystalline.

**Fig. 9:**
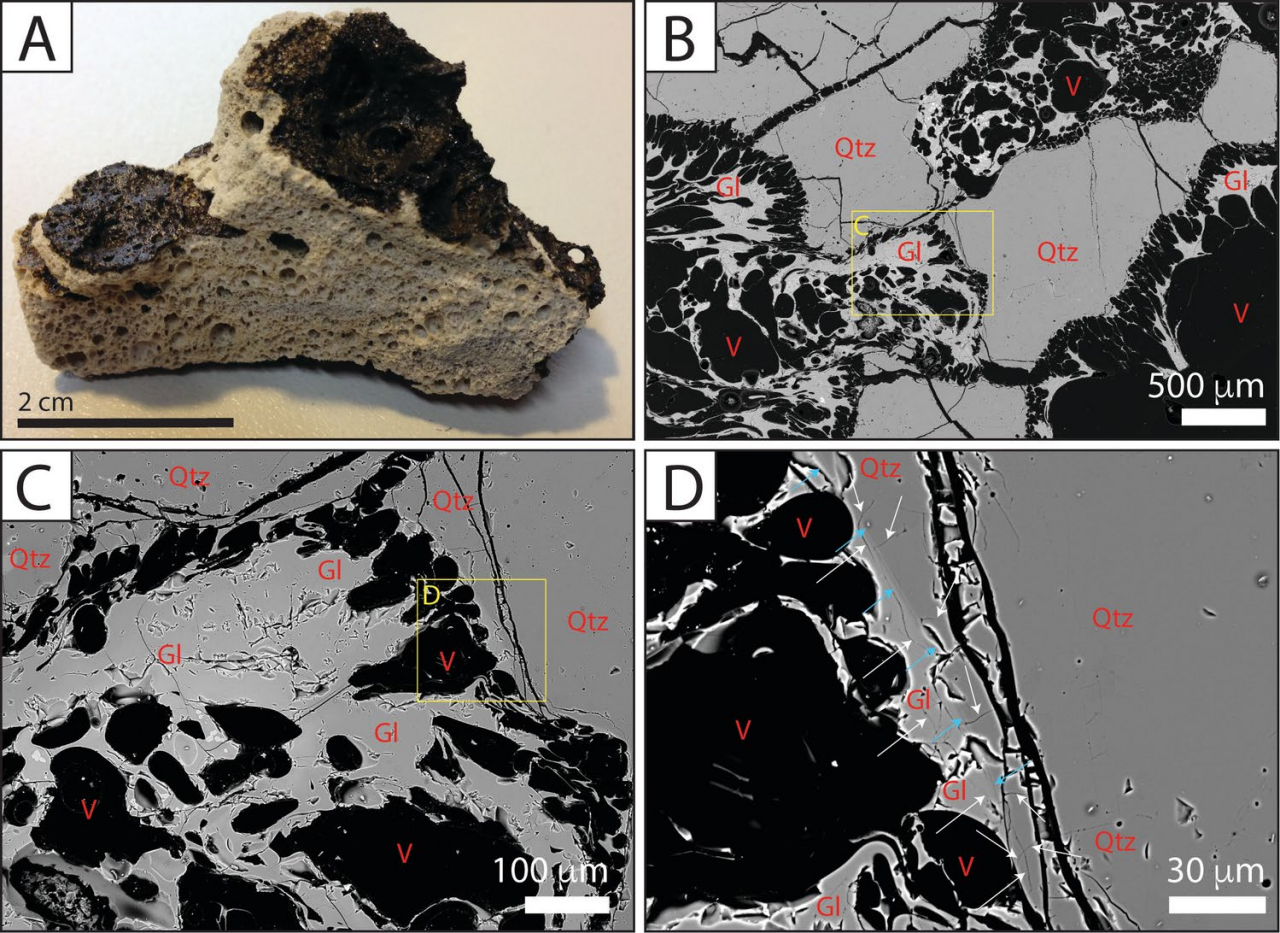
Example of mingled scoria found within volcaniclastic deposits at Little Mount quarry (sample MEC49Ai). **A** Hand specimen photograph, showing white/beige pumice mingled with black, vesicular basalt glass. **B**–**D** BSE images at increasing magnification (Qtz—quartz, Gl—glass, V—vesicle). The blue arrows indicate a sharp greyscale change at the quartz-glass interface, and the white arrows indicate orthogonal microfracture sets at the boundary (parallel to the boundary in the glass, and perpendicular in the quartz)

The quartz-rich, highly vesicular clasts mingled with basaltic scoria (Fig. 9A) were found dominantly in the Little Mount Quarry (Units LM2 and LM3). BSE images show anhedral/subhedral quartz macrocrysts suspended within a crystal-poor, highly vesicular (50–80%) glass. The vesicles at the quartz margin are higher in frequency, smaller and elongate (Fig. 9B,C). The boundary between the glass and quartz crystal is sharp and indicated by a grey-scale change in BSE imaging (Fig. 9D). Microcracks in the quartz grains are orthogonal to the contact and interrupted at the contact itself, whereas microcracks in the glass are predominantly parallel to the contact (Fig. 9D). Minor quartz (up to 5%) was also observed in thin sections from other samples (MEC7B, MEC64A and MEC45).

**Fig. 10:**
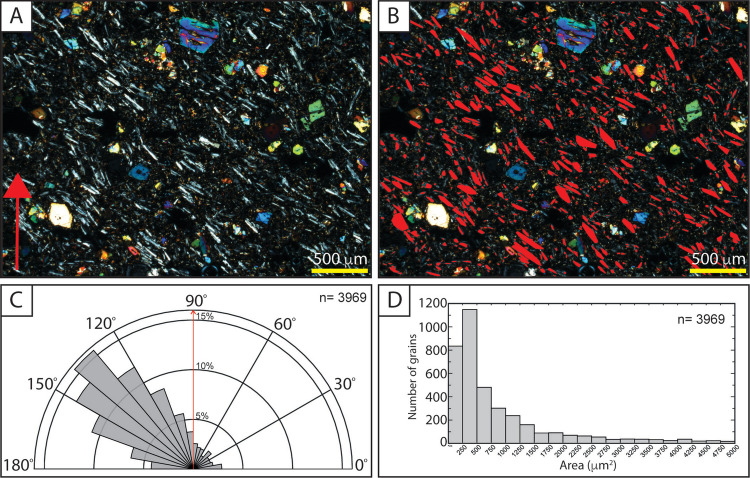
Crystal alignment within a dyke sample from Little Mount (MEC45). **A** Representative XPL photomicrograph at 25× magnification (red arrow indicates the long edge direction of the thin section, which is parallel to the N-S direction of the microscope, **B** same image as (**A**) but with detected plagioclase crystals indicated in red, **C** a unidirectional rose diagram of all plagioclase crystal orientations, with the red arrow from panel (**A**) indicated, and **D** a histogram showing the number of grains included in the fabric analysis in (**C**) and their size plotted as area

The dyke sample from the Little Mount Quarry (MEC45) has a trachytic texture (Fig. 10A,B), with a shape preferred orientation of plagioclase microlites (Fig. 10C,D). The crystal alignment observed may be caused by magma flow within a dyke or dyke segment that almost certainly fed a scoria cone in the Little Mount Quarry area.

### Geochemical trends

**Fig. 11:**
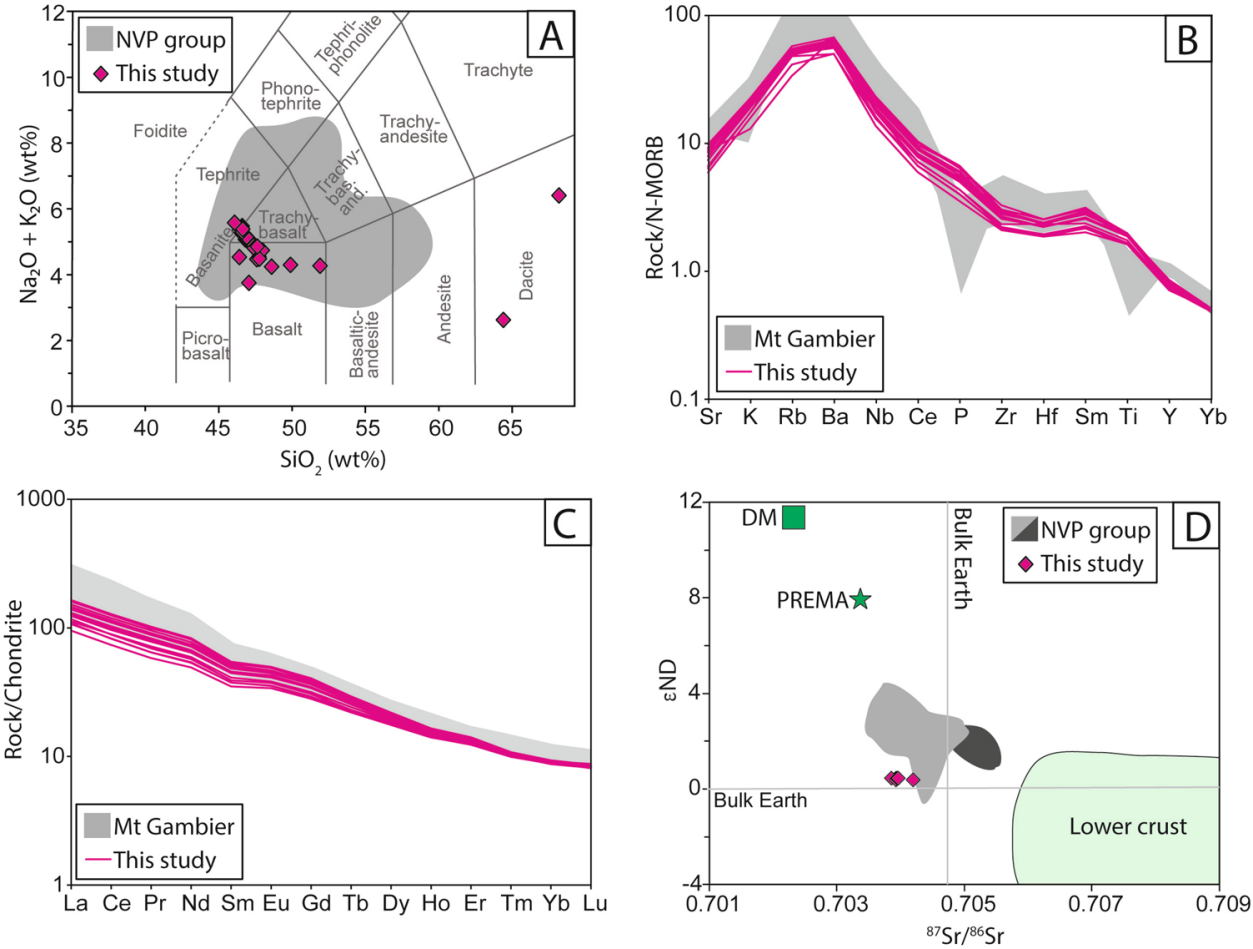
Geochemical data from the BBVC compared with other NVP products. **A** Total alkali-silica plot after Le Maitre (1984) compared with representative whole rock compositions across the NVP from Cas et al. (2017). **B** N-MORB-normalized trace element spider plot and **C** chondrite-normalized rare earth element plot (Sun and McDonough 1989) comparing BBVC data from this study with published values from the Mount Gambier Volcanic Province from Van Otterloo et al. (2014). DM = depleted mantle, PREMA = primitive mantle array. **D** Radiogenic Sr and Nd plot showing new BBVC data from this study and dark/light grey fields show all sub-alkaline plains samples and the alkaline cone field of the NVP, respectively (from Cas et al. (2017))

The whole rock geochemical measurements show the juvenile samples are basalt to trachybasalt in composition (Fig. 11A) and have Mg# between 61.6 and 65.2 (Table 1). They are subalkaline (Na_2_O + K_2_O totals range between 3.71 and 5.55 wt.%) and overlap with published data from other NVP volcanic centres (Fig. 11A). There is no correlation between the total alkalis and degree of evolution (Mg# or SiO_2_ concentration) in the BBVC samples. A lithic clast sample (MEC53D) has dacitic composition (Fig. 11A) with accompanying lower MgO concentration and variable total alkalis (Table 3). The mingled scoria sample (MEC49C) has dacitic compositions (Fig. 11A) with accompanying lower MgO concentrations and variable total alkalis (Table 3).

**Table 3:** Major element geochemistry of select samples across the BBVC. ^*^Repeat measurements of samples from Trowbridge (2003)

Sample	SiO_2_ (wt. %)	TiO_2_ (wt. %)	Al_2_O_3_ (wt. %)	Fe_2_O_3_ (wt. %)	FeO (wt. %)	MnO (wt. %)	MgO (wt. %)	CaO (wt. %)	Na_2_O (wt. %)	K_2_O (wt. %)	P_2_O_5_ (wt. %)	Total (wt. %)	Mg#
MEC19	47.99	2.09	13.71	1.88	9.61	0.16	10.09	9.28	3.45	1.26	0.47	100.00	65.16
MEC31	47.62	2.08	13.84	1.94	9.89	0.17	10.10	9.45	3.18	1.26	0.47	100.00	64.55
MEC27	48.63	2.08	14.02	1.96	10.00	0.18	9.51	9.00	3.06	1.14	0.42	100.00	62.87
MEC33	47.09	2.13	14.14	1.96	9.99	0.17	10.50	9.80	2.77	0.94	0.51	100.00	65.19
39A^*^	47.79	2.39	13.72	1.95	9.97	0.16	9.85	9.12	3.07	1.39	0.59	100.00	63.78
55F^*^	47.66	2.49	13.90	1.99	10.14	0.17	9.53	8.64	3.37	1.45	0.67	100.00	62.61
MEC4	49.91	2.23	13.59	1.87	9.52	0.16	9.15	8.79	3.01	1.26	0.51	100.00	63.13
MEC7	51.92	1.91	14.93	1.64	8.35	0.14	7.53	9.00	3.47	0.77	0.34	100.00	61.64
MEC7A	47.40	2.40	13.65	1.96	9.99	0.17	10.01	8.91	3.49	1.41	0.59	100.00	64.10
MEC7B	47.39	2.41	13.81	1.96	9.99	0.17	9.84	9.02	3.43	1.37	0.61	100.00	63.69
MEC9	47.76	2.52	14.04	2.01	10.23	0.17	9.55	8.54	3.13	1.38	0.67	100.00	62.45
MEC45	46.92	2.41	13.85	1.98	10.07	0.17	9.71	9.13	3.65	1.48	0.63	100.00	63.21
MEC49	46.84	2.47	13.83	2.00	10.20	0.17	9.72	9.03	3.46	1.58	0.70	100.00	62.95
MEC49C	68.17	0.90	16.98	0.72	3.66	0.04	1.58	1.38	2.53	3.85	0.19	100.00	43.51
MEC51	47.03	2.43	13.81	1.98	10.12	0.17	9.70	9.09	3.54	1.50	0.64	100.00	63.07
MEC53	46.91	2.47	13.81	1.99	10.15	0.17	9.64	9.08	3.49	1.59	0.70	100.00	62.86
MEC53D	64.38	1.16	14.22	1.40	7.12	0.10	4.36	3.95	1.06	1.53	0.72	100.00	52.18
MEC55	46.93	2.47	13.87	1.99	10.16	0.17	9.61	8.99	3.51	1.60	0.71	100.00	62.76
MEC55B	46.63	2.48	13.89	2.00	10.18	0.17	9.62	9.04	3.65	1.62	0.71	100.00	62.73
MEC59	46.10	2.51	13.78	1.99	10.17	0.17	9.77	9.14	3.84	1.71	0.81	100.00	63.12
MEC57	46.43	2.54	14.28	2.03	10.33	0.17	9.81	9.12	3.05	1.45	0.79	100.00	62.85
53^*^	46.66	2.52	13.76	2.00	10.19	0.17	9.56	9.01	3.71	1.64	0.78	100.00	62.56
MEC17	46.62	2.52	13.78	1.98	10.12	0.17	9.59	9.02	3.89	1.56	0.75	100.00	62.81
MEC18	46.61	2.54	13.70	2.00	10.21	0.17	9.50	9.10	3.79	1.60	0.79	100.00	62.39
MEC64A	46.41	2.47	13.79	2.01	10.23	0.17	9.87	8.98	3.68	1.63	0.76	100.00	63.22

There are no systematic differences in trace element concentrations across all BBVC samples analysed (Fig. 11B,C); however, coupled variations in Ni (210–246 ppm) indicate these melts have evolved since generation in the mantle (Table 4). The BBVC trace element data are like the general trends from the Mount Gambier Volcanic Province and have enrichments in LREE and the most incompatible elements compared with plain basalts of the NVP identified by Cas et al. (2017) (both transitional and subalkaline trends). Trace element ratios from the BBVC (Th/Yb and Nb/Yb) all plot within the MORB-OIB array of Pearce (2008), suggesting that no significant crustal contamination has occurred to these partial melts (see Supplementary Table S3). La/Yb ratios vary between 14.6 and 24.6 and Th/U ratios range between 3.8 and 6.6, with no correlation between degree of evolution (Mg#) and source characteristics (La/Yb and Th/U) (see Supplementary Table S3), suggesting that evolution is not coupled to changing source dynamics and instead may reflect varying amounts of minor fractionation and/or limited crustal assimilation.

**Table 4:** Key trace element proportions in select samples across the BBVC (for the full trace element list, please refer to the Electronic Appendix)

Sample	Ni (ppm)	Sr (ppm)	La (ppm)	Gd (ppm)	Hf (ppm)	Th (ppm)	U (ppm)
MEC19	237.6	579.7	25.7	6.0	3.9	3.0	0.7
MEC31	232.0	581.5	25.3	6.0	3.9	2.9	0.6
MEC27	220.6	537.0	22.5	5.8	3.9	2.5	0.5
MEC33	242.2	796.6	27.7	6.3	4.0	3.3	0.7
MEC4	216.9	602.7	26.5	6.5	4.8	4.9	0.7
MEC7A	245.9	669.5	29.4	7.0	4.7	3.2	0.8
MEC7B	236.0	675.5	29.8	7.0	4.7	3.1	0.8
MEC9	225.1	726.2	32.9	7.5	5.0	3.4	0.8
MEC45	230.3	717.1	30.6	7.1	4.6	3.0	0.8
MEC49	224.4	767.2	33.7	7.5	4.9	3.2	0.8
MEC51	227.1	718.8	31.1	7.1	4.6	3.0	0.8
MEC53	220.6	779.1	34.5	7.5	4.9	3.3	0.8
MEC55	218.6	777.1	34.6	7.6	4.8	3.2	0.8
MEC55B	216.7	777.8	34.4	7.5	4.9	3.2	0.8
MEC57	218.2	822.7	36.1	7.8	5.0	3.4	0.9
MEC17	213.6	843.2	37.9	8.1	5.3	3.9	1.0
MEC18	217.1	831.7	38.1	8.1	5.2	3.7	0.9
MEC64A	224.3	892.1	39.0	8.3	5.3	3.6	0.9

Radiogenic isotope data for the BBVC overlap the range of published ^87^Sr/^86^Sr ratios for the NVP (Cas et al. 2017) but extend to lower εNd for the lowest values of ^87^Sr/^86^Sr measured (Fig. 11D). The values of εNd are close to the bulk Earth value. Pb isotopes from the BBVC overlap with existing fields of data from alkaline cone basalts from the wider NVP (Cas et al. 2017), falling within the generalised subcontinental lithospheric mantle (SCLM) field.

## Discussion

### Origin of the lithic clasts

The lithic clasts identified at Porter’s Pit and the Little Mount Quarry include clay, sandstone, limestone, mafic plutonic lithics (pyroxenite) and a quartz-rich, highly vesicular material mingled with basaltic scoria. By comparing their compositions with the known lithostratigraphy from borehole data (Ryan et al. 1995; Gallagher and Holdgate 2000), the depths of volcanic explosions and how these evolved during the eruption can be determined (e.g. Van Otterloo et al. 2013).

Phreatomagmatic activity in the NVP has been attributed to magma interacting with aquifers, of which there are several possible candidates. The shallow aquifer system includes the Miocene Port Campbell Limestone, which extends to ~82 m depth at a borehole near Macarthur (Ryan et al. 1995), and the Clifton Formation which is limestone at ~180–210 m depth. The limestone lithic clasts therefore suggest shallow explosions (< 200 m), consistent with expected explosion depths for phreatomagmatic eruptions (Valentine et al. 2014).

Magma interaction with potentially deeper aquifers includes the Palaeocene Dilwyn Formation, which is a confined coarse-grained sandstone aquifer located ~ 250–285 m depth (Ryan et al. 1995), and the Palaeocene Pebble Point Formation at ~ 385–320 m depth (Ryan et al. 1995), comprising coarse-grained sandstones and conglomerates with well-rounded quartz pebbles. These are likely candidates for the sandstone lithic clasts and indicate deeper explosions, though these would be expected to be higher energy explosions due to the increased source depth (~200 to 500 m) (Valentine et al. 2014).

The Miocene Gellibrand Marl could be a potential shallow source of erupted silty clay and marl lithic clasts, sitting at ~80–180 m depth between the Port Campbell Limestone and Clifton Formation. However, deeper sources could also include mudstone and siltstone beds within the Dilwyn Formation (~250–285 m depth). The large clay clasts were likely the shallowest lithic clasts included in the deposits, as they appeared very similar to the clay unit documented at the base of the sequences exposed at Porter’s Pit and Little Mount Quarry (Figs. 6 and 7).

The origin of the mafic plutonic lithic clasts found within the Porter’s Pit deposits is ambiguous; however, they could derive from the BBVC magma intrusive network (van den Hove et al. 2017a). The Cretaceous Eumeralla Formation comprises volcaniclastic basement rocks located at ~ 320–750 m depth (Ryan et al. 1995)—this aquitard likely marks the deepest levels of phreatomagmatic explosions shallower than 320 m depth.

The quartz-rich mingled scoria clasts have an appearance and vesicularity that is similar to a rhyolitic pumice; however, there are currently no silica-rich eruptions identified within the NVP, and so alternative explanations for their origin are needed. They have similar clast morphology to restingolites (Troll et al. 2011), which have a core of white and porous pumice-like material and were some of the first materials ejected during the 2011 eruption of El Hierro, Canary Islands. Those clast textures, petrography and geochemical compositions confirmed the clast cores were xenolithic in origin, leading to them being called xenopumices (Meletlidis et al. 2012). Silica-rich xenoliths were broken off from the wall rock by the ascending dyke, and the heat from the magma causes them to partially melt and vesiculate. Similar examples of xenolith-derived pumice have also been described in eruptions from Parícutin, Mexico (Milton 1944), Izu Peninsula in Japan (Yamamoto et al. 1991), the Grímsnes craters and Surtsey in Iceland (Jakobsson 1966; Sigurdsson 1968), the Auvergne (France), Eifel (Germany), Mt Melbourne in Antarctica (Burchardt et al. 2016) and Göllüdağ volcanic centre in Turkey (Akin et al. 2024). If the Budj Bim quartz-rich mingled scoria clasts are xenopumice, this would make them the first identified xenopumices in Australia, as far as we are aware.

The eruption of xenopumice (Troll et al. 2011; Meletlidis et al. 2012) suggests partial assimilation of quartz-rich xenoliths has occurred. At temperatures > 800 °C, laboratory experiments have shown that sandstone can partially melt within a few hours (Lintao et al. 2017). The basalt magma erupted at Budj Bim would have likely ascended at temperatures ~1200 °C, and so the partial preservation of these xenoliths might suggest rapid magma ascent or large grain sizes. Deeply sourced rounded quartz pebbles (Pebble Point Formation, ~285–320 m depth), basement granite (> 760 m depth) or vein quartz could be potential source materials.

### A new proposed eruptive history of the Budj Bim Volcanic Complex

Our results support past suggestions that the BBVC comprises at least ten inferred eruption points that are associated with key volcanic structures (the fissure crater, scoria cones and spatter cones; Fig. 3) (Edney 1987; Tunjc 1988; Trowbridge 2003). We have found no evidence of significant time breaks in the stratigraphy at Budj Bim, as the brown ash layers we described in the scoria cone deposits at Porter’s Pit Quarry and Little Mount Quarry are not due to surface weathering but due to hydration and palagonitisation of already wet phreatomagmatic glassy ashes (Stroncik and Schmincke 2002). This suggests the eruption was broadly continuous, with perhaps short breaks occurring intermittently. With Mg# below 70, the BBVC magmas are not primitive mantle melts, and small degrees of evolution are likely to have occurred at depth. The data suggest that fractionation of minor olivine, shown by the coupled Mg# and Ni concentration changes, must have occurred prior to the BBVC melts being tapped during eruption.

We have reconstructed the eruptive history of the BBVC and explain this through five key phases: 1—magma generation and ascent, 2—fissure eruption and magma fountaining, 3—eruption point migration, phreatomagmatism and the Little Mount scoria cone, 4—building the Mount Eccles scoria cone and 5—magma fountaining and breach of a lava lake.

#### Phase 1: magma generation and ascent

Tectonic setting and crustal architecture are known to strongly impact the dynamics of magma ascent through the crust, controlling the location of eruptions, ascent rates and the tendency for magmas to stall or have a direct pathway to the surface (Burchardt et al. 2022). Magma ascent would have begun with a basaltic dyke intersecting the Yarramyljup Fault (Chukwu et al. 2025) and a branch of the Ardonachie Regional Fault (Vujovic et al. 2021) at several kilometres depth. The BBVC is bound to the west by the Yarramyljup and to the east by the Hummocks N-S trending fault systems of Neoproterozoic-Cambrian age. Between these faults, reactivated in the Mesozoic, lies a dense network of NW-SE normal faults, detected by geophysical methods, which developed during rifting in the Late Cretaceous (Chukwu et al. 2025). These structures are mostly buried beneath Cretaceous to Miocene passive margin sedimentary sequences of the Otway Basin (Finlayson et al. 1996; Bryan et al. 1997) and intraplate volcanic successions of the NVP. NW-SE and N-S trending faults, striking parallel to horizontal σ_1_, served as conduits for magma ascent along dykes, which, in these settings, would have otherwise stalled in the crust as sills (van den Hove et al. 2017a, b). Dyke segments oriented NNE (~60° offset from the fissure trend) are instead related to stress deviations in the shallow crust. The BBVC developed in proximity to, but separately from, an unnamed NW-SE trending fault. It is likely that during ascent, the magma exploited a similar neighbouring, but unrecognised, NW-SE fault.

**Fig. 12:**
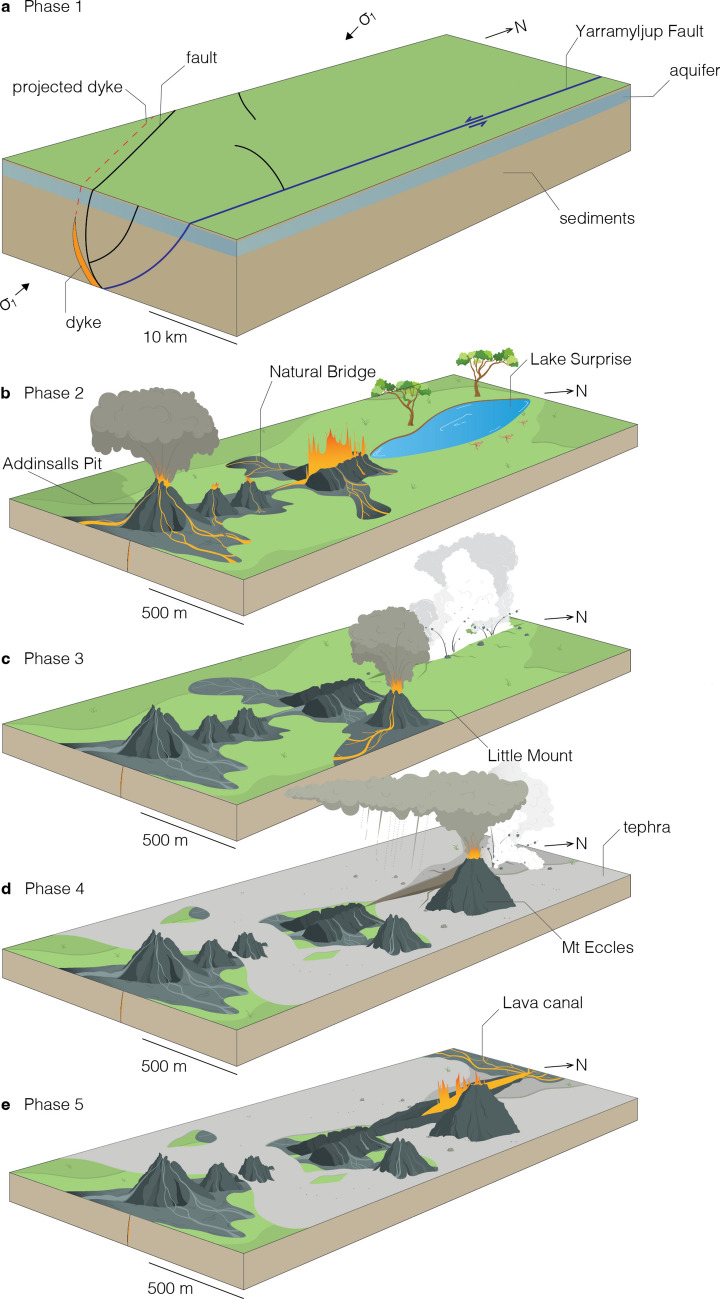
Schematic diagrams depicting the five phases of magma ascent and eruption of the Budj Bim Volcanic Complex (scale bar for illustrative purposes only). **A** Phase 1: large-scale view of magma ascent during Quaternary compressional tectonics, showing faults (thick black lines), projected Budj Bim dyke path and shallow aquifers. **B** Phase 2: zoomed-in view of early fissure eruption and magma fountaining. **C** Phase 3: eruption point migration with phreatomagmatism and violent Strombolian/micro-Plinian activity. **D** Phase 4: micro-Plinian eruption at Mount Eccles with intermittent phreatomagmatism. **E** Phase 5: magma fountaining and breach of lava lake to feed lava-tube-fed lava flows

Prior to eruption, the landscape would have been broadly flat-lying, with mud/clay-dominated swamp deposits at the surface (Fig. 12A). It is possible that some earthquakes may have been felt at the surface in the days, or perhaps weeks, before the eruption. However, similar recent events in Iceland (e.g. 2021) have indicated reduced amounts of host rock deformation (e.g. surface elevation changes, the number and size of earthquakes) in the lead up to an eruption when dykes intrude regional faults (Sigmundsson et al. 2022).

#### Phase 2: fissure eruption and magma fountaining

The eruption began as a linear fissure, extending from the most southerly point of Addinsalls Pit to the Little Mount Dry Crater (~1 km long, Fig. 12B). Magma fountaining along the fissure transitioned to violent Strombolian or micro-Plinian activity (LM1 and PP1) at Addinsalls Pit. Some erupted clasts were re-incorporated into the magma fountains to produce cored and armoured lapilli near the Little Mount Dry Crater. A linear spatter rampart formed, and the eruption quickly localised to form spatter cones (The Shaft, The Pit and The Alcove) as lava flows from reconstituted spatter flowed away from these eruption points, producing the Natural Bridge lavas, small flows exposed in the Little Mount Quarry and the lavas at Addinsalls Pit.

Phase 2 was a sustained, high effusive magmatic phase early in the eruption; the spatter ridge and lava inundation would have appeared very similar to that produced during the eruptions in Iceland, such as Bárðarbunga 2014 (Sigmundsson et al. 2015), Fagradalsfjall 2021 and Svartsengi 2023–2025 (Sigmundsson et al. 2024). These eruptions saw the opening of fissures and the beginnings of spatter cone formation happen within a few hours of eruption onset, with scoria cones developing within the first few days. We suggest that a similar timescale would be highly likely for the BBVC.

#### Phase 3: eruption point migration, phreatomagmatism and the Little Mount scoria cone

Phase 3 saw the start of phreatomagmatism due to fissure lengthening ~1.2 km to the NNW into the Lake Surprise area (Fig. 12C). Lithic clast–rich deposits at Porter’s Pit were produced with low amounts of juvenile material (Units PP2 and PP3), relatively high proportion of coarse ash and fragmented clasts, with phreatomagmatic activity likely due to interaction with an aquifer that was periodically recharged at Lake Surprise. We infer that the Lake Surprise volcanic fissure crater was created by explosions from depths upwards of 600 m eroding out country rock fragments from underlying Palaeogene, Neogene and Quaternary sedimentary strata and Older Volcanic units (Gallagher and Holdgate 2000). The scalloped margin of Lake Surprise suggests three different eruption points were active, and depositional structures suggest the generation of discrete pyroclastic surges from the Lake Surprise crater. This activity is different to Iceland analogues where phreatomagmatism is relatively limited (Thordarson and Larsen 2007). Surge deposits are generally less likely to be preserved in volcanic terrains, as evidenced by minimal deposits left by significant phreatic and phreatomagmatic eruptions at Whakaari/White Island, New Zealand (Kilgour et al. 2019). This type of volcanic activity is therefore under-recorded and consequently under-recognised; however, at Budj Bim, the close temporal relationship of the surge generation with magmatic activity, and intercalation of their deposits with scoria fallout, has assisted in their preservation.

Little Mount Quarry deposits record violent Strombolian to micro-Plinian activity at this time, building up a scoria cone at Little Mount (LM2, LM3 and LM4 deposits). However, this site also exhibits two eruption points simultaneously active, spaced ~600 m apart, with very different eruptive styles, as lithic clasts within these deposits likely originate from phreatomagmatism at Lake Surprise. The preservation of partially assimilated quartz-rich xenoliths within mingled basalt scoria magmas ascended from > 1 km depth, potentially within 1 h, corresponding to an estimated magma ascent rate of 0.3 m/s, which is moderately rapid (Jones et al. 2018).

#### Phase 4: building the Mount Eccles scoria cone

In Phase 4, the eruption progressed to an intense period of point-focused magmatic explosivity, with a micro-Plinian sustained column building up the Mount Eccles scoria cone, punctuated by intermittent spatter bursts (Fig. 12D). Diffuse stratification in the deposits (PP4 and PP6) represents gentle pulsing in eruptive intensity. Magmatism was interrupted by brief phreatomagmatism from the Lake Surprise fissure crater, as magma periodically interacted with lake water, generating pyroclastic surges (PP5). These phreatomagmatic eruptions created a blanket of ash across the BBVC (LM5) and the micro-Plinian column deposited fallout at the Little Mount Quarry area (LM6).

#### Phase 5: magma fountaining and breach of a lava lake

The last phase of the BBVC eruptions saw sustained magma fountaining at Lake Surprise with the formation and breach of a lava lake (Fig. 12E). Magma-fountaining explosions truncated the Little Mount scoria cone to the south, producing densely welded agglutinated spatter layers producing lava flows at high topography due to magma fountaining fallout. Towards the middle of the fissure crater, in situ agglutinated spatter from magma fountaining in the fissure laterally transformed into clastogenic lava; some of these lavas overspilled the crater, whilst others flowed back into the crater. A lava lake within the fissure then breached to the north at Lava Canal feeding passive outpourings which spread widely to the east and west (Ollier 1964a; Grimes 1995; Trowbridge 2003) and then > 30 km to the south as the Tyrendarra lava flow.

### Comparing basaltic fissure eruptions in marine basaltic island settings and continental settings

Many studies of fissure vent eruptions in oceanic plate settings such as Iceland have demonstrated that phreatomagmatic eruptions are relatively rare (Thordarson and Larsen 2007). Most are largely magmatic (e.g. 2014–2015 Bárðarbunga-Holuhraun fissure eruption, Pedersen et al. 2017), represented by lava flows and spatter and scoria cones, although explosive phreatomagmatic interactions through surface lakes, thick snow and ice can occur as fissure vent systems propagate laterally (e.g. Mattsson and Höskuldsson 2011). The deposits of such phreatomagmatic eruptions are dominated by juvenile basaltic shards and accretionary aggregates, and near-surface basaltic lithic pyroclasts, transported by fallout and base surge processes (Mattsson and Höskuldsson 2011).

In contrast, in continental settings where intraplate volcanic provinces form and eruptions occur through aquifer-bearing sedimentary basin successions (e.g. the southern half of the NVP), many volcanoes, including fissure vent systems, experience varying degrees of phreatomagmatic eruption activity (Cas et al. 2017). At one extreme, the ~5 ka Mt Gambier Volcanic Complex consists of a WNW alignment of maar craters and spatter and scoria cones along a 3 km long fissure vent system. 60% of its deposits are phreatomagmatic base surge and fallout deposits, containing a mixture dominated by crustal derived xenoliths of older limestone, sandstone and mudstone, and abundant quartz xenocrysts derived from sedimentary substrate, including the triggering subsurface aquifers, as well as lesser juvenile basaltic debris (Van Otterloo et al. 2013).

The BBVC is dominated by magmatic eruption deposits of lavas and spatter and scoria cones, with only a minor component of phreatomagmatic eruption deposits in Phase 3 and 4 sequences (Fig. 12). Those deposits also bear the mark of explosive interaction between rising basaltic magma and aquifers within a continental crustal sequence, with some pyroclastic debris sourced from terrigenous sedimentary aquifers up to 600 m below the Earth’s surface, which does not occur in marine basaltic island settings.

## Conclusions

We have proposed a new eruptive history for the Budj Bim Volcanic Complex based on information gathered from the volcanic deposits, structures, petrology and geochemistry. We suggest the Budj Bim eruption can be divided into five key phases:

- Phase 1 involves magma generation and ascent. The whole rock geochemical results show the erupted products are basalt to trachybasalt and not primitive melts with Mg# < 70. They are subalkaline in composition and trace element ratios show no significant crustal contamination has occurred. Radiogenic isotope data overlap with the range of ^87^Sr/^86^Sr ratios of the NVP, with Pb isotopes falling within the generalised subcontinental lithospheric mantle field. Magma ascent would have been rapid and facilitated by a dyke intersecting the major Yarramyljup Fault at depth and branches of the Ardonachie regional faults at shallower levels.
- Phase 2 is when the fissure eruption began, with magma fountaining stretching from Addinsalls Pit to the Little Mount Dry Crater, building a linear spatter rampart. A small scoria cone formed at Addinsalls Pit through micro-Plinian activity, followed by a row of spatter cones (The Alcove, The Pit, The Shaft) and several clastogenic lava flows.
- In phase 3, the fissure lengthened and extended into the Lake Surprise area. This caused phreatomagmatism, with explosions creating a linear fissure crater. Simultaneously, a scoria cone built up due to violent Strombolian to micro-Plinian activity at a new eruption point offset from the fissure trend and located at Little Mount Quarry. This erupted ‘mingled pumice’, which we interpret, to the best of our knowledge, to be the first description of xenopumice in Australian volcanics.
- Phase 4 saw the construction of the Little Mount scoria cone with micro-Plinian activity. This magmatic explosivity is interrupted with periods of phreatomagmatism from Lake Surprise, which produces pyroclastic surges and covers the whole area in ash.
- Phase 5 is the culmination of the eruption, with magma fountaining occurring across the three Lake Surprise eruption points; explosions at the southerly point truncated the Mount Eccles scoria cone, and spatter agglutinated at high levels. Clastogenic lavas overspilled adjacent to the central eruption point, with some draining back into the crater. A lava lake breached from the north of the elongate crater and passively filled the land with lava. This spread east, west and then south to feed the substantial Tyrendarra lava flow, stretching more than 30 km to the south coast.

The Budj Bim eruption would have been like the 2014–2015 Bárðarbunga-Holuhraun fissure eruption in Iceland, which had similar eruptive processes, erupted volume, and, we expect, similar duration (approximately 6 months). However, Budj Bim had a greater variety of explosive eruption styles, with notable phreatomagmatism alongside significant magmatic explosivity and lava flow generation. This distinction could be due to the continental, rather than oceanic, setting of Budj Bim and greater interaction with the substrate. The Budj Bim eruption is therefore an excellent example of how fissure eruptions in continental settings are more complex than is often assumed and have additional dangers and impacts that need to be taken into consideration in hazard assessments.

## Supplementary Information

Below is the link to the electronic supplementary material.

ESM 1 (DOCX 30.6 MB)

ESM 2 (DOCX 28.1 KB)

ESM 3 (DOCX 28.1 KB)

ESM 4 (XLSX 43.0 KB)

ESM 5 (DOCX 75.0 KB)

## Data Availability

All data supporting the findings of this study are available within the paper and its Supplementary Information.

## References

[CR1] Akin L, Aydar E, Ceylan A (2024) A deep insight into a chemically homogeneous banded pumice sample: a role of crystal cargo immiscibility. Turk J Earth Sci 33:341–361. 10.55730/1300-0985.1915

[CR2] Australian Bureau of Statistics 2023–24 (2025) Regional population. In: https://www.abs.gov.au/statistics/people/population/regional-population/latest-release. Accessed 28 Mar 2026

[CR4] Bishop MA (2007) Point pattern analysis of eruption points for the Mount Gambier volcanic sub‐province: a quantitative geographical approach to the understanding of volcano distribution. Area 39:230–241.

[CR5] Blackburn G, Allison GB, Leaney FWJ (1982) Further evidence on the age of the tuff at Mt Gambier, South Australia. Trans R Soc S Aust 106:163–167

[CR6] Blaikie TN, van Otterloo J, Ailleres L et al (2015) The erupted volumes of tephra from maar volcanoes and estimates of their VEI magnitude: examples from the late Cenozoic Newer Volcanics Province, south-eastern Australia. J Volcanol Geotherm Res 301:81–89. 10.1016/j.jvolgeores.2015.05.004

[CR7] Boyce J (2013) The newer volcanics province of southeastern Australia: a new classification scheme and distribution map for eruption centres. Aust J Earth Sci 60:449–462. 10.1080/08120099.2013.806954

[CR8] Boyce JA, Nicholls IA, Keays RR, Hayman PC (2015) Variation in parental magmas of Mt Rouse, a complex polymagmatic monogenetic volcano in the basaltic intraplate Newer Volcanics Province, southeast Australia. Contributions to Mineralogy and Petrology. 10.1007/s00410-015-1106-y

[CR9] Brown T (2024) Recognising the Budj Bim cultural landscape as world heritage: how a socio-material approach bridged the tangible-intangible heritage gap. Aust J Adult Learn 64:33–52

[CR10] Bryan SE, Constantine AE, Stephens CJ et al (1997) Early Cretaceous volcano-sedimentary successions along the eastern Australian continental margin: implications for the break-up of eastern Gondwana. Earth Planet Sci Lett 153:85–102

[CR11] Burchardt S, Troll VR, Schmeling H et al (2016) Erupted frothy xenoliths may explain lack of country-rock fragments in plutons. Sci Rep. 10.1038/srep34566 10.1038/srep34566 PMC5090209 27804996

[CR12] Burchardt S, Annen CJ, Kavanagh JL, Hilmi Hazim S (2022) Developments in the study of volcanic and igneous plumbing systems: outstanding problems and new opportunities. Bull Volcanol. 10.1007/s00445-022-01564-6

[CR13] Cas RAF, van Otterloo J, Blaikie TN, van den Hove J (2017) The dynamics of a very large intra-plate continental basaltic volcanic province, the Newer Volcanics Province, SE Australia, and implications for other provinces. In: Geological Society Special Publication. Geological Society of London, pp 123–172

[CR14] Chukwu C, Betts P, Munukutla R et al (2025) A new approach to imaging deep crustal structures: implications for the crustal architecture of southeast Australia’s passive margin. J Geophys Res Solid Earth. 10.1029/2024JB029974

[CR15] Delbrel J, Burton M, Engwell S et al (2025) An investigation of changes to commercial aircraft flight paths during volcanic eruptions. J Appl Volcanol. 10.1186/s13617-025-00150-7

[CR16] Demidjuk Z, Turner S, Sandiford M et al (2007) U-series isotope and geodynamic constraints on mantle melting processes beneath the Newer Volcanic Province in South Australia. Earth Planet Sci Lett 261:517–533. 10.1016/j.epsl.2007.07.006

[CR17] Dharmarathna B (2022) Holocene hydroclimate variability in south-eastern Australia; validation and application of cellulose oxygen isotopes at Lake Surprise, Victoria. University of Adelaide, Australia.

[CR19] Edney WJ (1987) Facies contrasts and their origins in scoria cones, maars and tuff rings of the Newer Volcanics Province, Western Victoria, Australia. Masters Thesis, Monash University, pp. 124

[CR20] Finlayson DM, Johnstone DW, Owen AJ, Wake-Dyster KD (1996) Deep seismic images and the tectonic framework of early rifting in the Otway Basin, Australian southern margin. Tectonophysics 264:137–152

[CR21] Foden J, Elburg MA, Dougherty-Page J, Burtt A (2006) The timing and duration of the delamerian orogeny: correlation with the ross orogen and implications for Gondwana assembly. J Geol 114:189–210

[CR22] Foote A, Németh K, Handley H (2022) The interplay between environmental and magmatic conditions in eruption style transitions within a fissure-aligned monogenetic volcanic system of Auckland, New Zealand. Journal of Volcanology and Geothermal Research. 10.1016/j.jvolgeores.2022.107652

[CR23] Foster DA, Gleadow AJW (1992) Reactivated tectonic boundaries and implications for the reconstruction of southeastern Australia and northern Victoria Land, Antarctica. Geology. 10.1130/0091-7613(1992)020<0267:rtbaif>2.3.co;2

[CR24] Fraser S, Soto-Berelov M, Holden L et al (2025) Mapping young lava rises (stony rises) across an entire basalt flow using remote sensing and machine learning. Remote Sens. 10.3390/rs17122004

[CR25] Gallagher SJ, Holdgate G (2000) The palaeogeographic and palaeoenvironmental evolution of a Palaeogene mixed carbonate-siliciclastic cool-water succession in the Otway Basin, Southeast Australia. Palaeogeogr Palaeoclimatol Palaeoecol 156:19–50

[CR26] Gernon TM, Brown RJ, Tait MA, Hincks TK (2012) The origin of pelletal lapilli in explosive kimberlite eruptions. Nat Commun. 10.1038/ncomms1842 10.1038/ncomms1842 22588294

[CR27] Gill ED (1979) The Tyrendarra lava flow, Western Victoria, Australia. Victorian Nat 96:227–229.

[CR28] Giordano G, Cas R, Wright JV (2024) Explosive eruption styles, columns, and pyroclastic fallout deposits. In: Volcanology. Springer Textbooks in Earth Sciences, Geography and Environment. Springer, Cham. 10.1007/978-3-319-66613-6_9

[CR29] Gray CM, McDougall I (2009) K-Ar geochronology of basalt petrogenesis, Newer Volcanic Province, Victoria. Aust J Earth Sci 56:245–258.

[CR30] Grimes K (1995) Lava caves and channels at Mount Eccles, Victoria. In: Proceedings of 20th Conference of the ASF. pp 15–22

[CR31] Grimes KG (2002) Small subcrustal lava caves: examples from Victoria, Australia. AMCS Bulletin 19/SMES Boletín 735–44

[CR32] Hamilton CW, Thordarson T, Fagents SA (2010) Explosive lava-water interactions i: architecture and emplacement chronology of volcanic rootless cone groups in the 1783–1784 Laki lava flow, Iceland. Bull Volcanol 72:449–467. 10.1007/s00445-009-0330-6

[CR33] Handley H, Van Otterloo J, Cas R (2018) Would an eruption in Melbourne really match Hawaii’s volcanoes? Here’s the evidence. In: The Conversation. https://theconversation.com/would-an-eruption-in-melbourne-really-match-hawaiis-volcanoes-heres-the-evidence-101675. Accessed 14 Jan 2026

[CR34] Hargitai H, Kereszturi Á (2015) Encyclopedia of planetary landforms. Springer.

[CR35] Hayes JL, Biass S, Jenkins SF et al (2022) Integrating criticality concepts into road network disruption assessments for volcanic eruptions. Journal of Applied Volcanology. 10.1186/s13617-022-00118-x

[CR36] Head JW, Wilson L (1989) Basaltic pyroclastic eruptions influence of gas-release patterns and volume fluxes on fountain structure, and the formation of cinder cones, spatter cones, rootless flows, lava ponds and lava flows. J Volcanol Geotherm Res 37:261–271

[CR37] Hjartardóttir ÁR, Dürig T, Parks M et al (2023) Pre-existing fractures and eruptive vent openings during the 2021 Fagradalsfjall eruption, Iceland. Bull Volcanol. 10.1007/s00445-023-01670-z

[CR38] Holm RF (1987) Significance of agglutinate mounds on lava flows associated with monogenetic cones: an example at Sunset Crater, northern Arizona. Geol Soc Am Bull 99:319–324.

[CR39] Horwell CJ, Baxter PJ (2006) The respiratory health hazards of volcanic ash: a review for volcanic risk mitigation. Bull Volcanol 69:1–24.

[CR40] Houghton BF, Tisdale CM, Llewellin EW et al (2021) The birth of a Hawaiian fissure eruption. J Geophys Res Solid Earth. 10.1029/2020JB020903

[CR41] Jakobsson S (1966) The grimsnes lavas SW-Iceland. Acta Naturalia Islandica. pp 5–30

[CR42] Jiang C, Yang Y, Rawlinson N, Griffin WL (2016) Crustal structure of the Newer Volcanics Province, SE Australia, from ambient noise tomography. Tectonophysics 683:382–392. 10.1016/j.tecto.2016.06.033

[CR44] Jones MP, Soule SA, Gonnermann HM et al (2018) Magma ascent and lava flow emplacement rates during the 2011 Axial Seamount eruption based on CO2 degassing. Earth Planet Sci Lett 494:32–41. 10.1016/j.epsl.2018.04.044

[CR45] Jordan SC, Cas RAF, Hayman PC (2013) The origin of a large (>3km) maar volcano by coalescence of multiple shallow craters: Lake Purrumbete maar, southeastern Australia. J Volcanol Geotherm Res 254:5–22. 10.1016/j.jvolgeores.2012.12.019

[CR46] Joyce B (1975) Quaternary volcanism and tectonics in southeastern Australia. In: Suggate RP, Cresswell MM (eds) Quaternary studies. The Royal Society of New Zealand, Wellington, pp 169–178.

[CR47] Joyce EB (1976) Lava channels and associated caves in Victoria, Australia. Proc Int Symposium on Vulcanospeleology and its extraterrestrial Applications 51–57

[CR48] Kilgour G, Gates S, Kennedy B et al (2019) Phreatic eruption dynamics derived from deposit analysis: a case study from a small, phreatic eruption from Whakāri/White Island, New Zealand. Earth, Planets and Space. 10.1186/s40623-019-1008-8

[CR49] Kósik S, Németh K, Kereszturi G et al (2016) Phreatomagmatic and water-influenced Strombolian eruptions of a small-volume parasitic cone complex on the southern ringplain of Mt. Ruapehu, New Zealand: facies architecture and eruption mechanisms of the Ohakune Volcanic Complex controlled by an unstable fissure eruption. J Volcanol Geotherm Res 327:99–115. 10.1016/j.jvolgeores.2016.07.005

[CR50] Le Maitre RW (1984) A proposal by the IUGS subcommission on the systematics of igneous rocks for a chemical classification of volcanic rocks based on the total alkali silica (TAS) diagram. Aust J Earth Sci 31:243–255. 10.1080/08120098408729295

[CR51] Lesti C, Giordano G, Salvini F, Cas R (2008) Volcano tectonic setting of the intraplate, Pliocene-Holocene, Newer Volcanic Province (southeast Australia): role of crustal fracture zones. Journal of Geophysical Research: Solid Earth. 10.1029/2007JB005110

[CR52] Lintao Y, Marshall AM, Wanatowski D et al (2017) Effect of high temperatures on sandstone - a computed tomography scan study. Int J Phys Model Geotech 17:75–90. 10.1680/jphmg.15.00031

[CR54] Magee C, Muirhead JD, Karvelas A et al (2016) Lateral magma flow in mafic sill complexes. Geosphere 12:809–841. 10.1130/GES01256.1

[CR56] Matchan EL, Phillips D, Jourdan F, Oostingh K (2020) Early human occupation of southeastern Australia: new insights from 40Ar/39Ar dating of young volcanoes. Geology 48:390–394. 10.1130/G47166.1

[CR57] Mattsson HB, Höskuldsson Á (2011) Contemporaneous phreatomagmatic and effusive activity along the Hverfjall eruptive fissure, north Iceland: eruption chronology and resulting deposits. J Volcanol Geotherm Res 201:241–252. 10.1016/j.jvolgeores.2010.05.015

[CR58] McNiven IJ, Crouch J, Richards T et al (2012) Dating Aboriginal stone-walled fishtraps at Lake Condah, southeast Australia. J Archaeol Sci 39:268–286. 10.1016/j.jas.2011.09.007

[CR59] Meletlidis S, Di Roberto A, Pompilio M et al (2012) Xenopumices from the 2011-2012 submarine eruption of El Hierro (Canary Islands, Spain): constraints on the plumbing system and magma ascent. Geophys Res Lett. 10.1029/2012GL052675

[CR60] Menand T, Daniels KA, Benghiat P (2010) Dyke propagation and sill formation in a compressive tectonic environment. Journal of Geophysical Research: Solid Earth. 10.1029/2009JB006791

[CR62] Miller JM, Norvick MS, Wilson CJL (2002) Basement controls on rifting and the associated formation of ocean transform faults-Cretaceous continental extension of the southern margin of Australia. Tectonophysics 359:131–155.

[CR63] Milton C (1944) Notes on volcanic rocks from Parícutin, Mexico. Trans Am Geophys Union 25:618–621. 10.1029/TR025i004p00618

[CR64] Murcia H, Németh K, El-Masry NN et al (2015) The Al-Du’aythah volcanic cones, Al-Madinah City: implications for volcanic hazards in northern Harrat Rahat, Kingdom of Saudi Arabia. Bull Volcanol. 10.1007/s00445-015-0936-9

[CR65] Németh K, Toni M, Sokolov V, et al (2024) Eruption scenarios of a monogenetic volcanic field formed within a structurally controlled basement terrain: Harrat Lunayyir, Saudi Arabia. In: A Comprehensive Study of Volcanic Phenomena. IntechOpen. 10.5772/intechopen.1008330

[CR66] Ollier CD (1964a) Caves and related features of Mount Eccles. Victorian Nat 81:64–71.

[CR67] Ollier CD (1964b) Tumuli and lava blisters of Victoria Australia. Nature 202:1284–1286.

[CR68] Ollier CD, Joyce EB (1964) Volcanic physiography of the western plains of Victoria. Proc R Soc Victoria 77:357.

[CR69] Oostingh KF, Jourdan F, Matchan EL, Phillips D (2017) 40Ar/39Ar geochronology reveals rapid change from plume-assisted to stress-dependent volcanism in the Newer Volcanic Province, SE Australia. Geochem Geophys Geosyst 18:1065–1089. 10.1002/2016GC006601

[CR70] Pallister JS, McCausland WA, Jónsson S et al (2010) Broad accommodation of rift-related extension recorded by dyke intrusion in Saudi Arabia. Nat Geosci 3:705–712. 10.1038/ngeo966

[CR72] Pearce JA (2008) Geochemical fingerprinting of oceanic basalts with applications to ophiolite classification and the search for Archean oceanic crust. Lithos 100:14–48. 10.1016/j.lithos.2007.06.016

[CR73] Pedersen GBM, Höskuldsson A, Dürig T et al (2017) Lava field evolution and emplacement dynamics of the 2014–2015 basaltic fissure eruption at Holuhraun, Iceland. J Volcanol Geotherm Res 340:155–169. 10.1016/j.jvolgeores.2017.02.027

[CR74] Price RC, Gray CM, Frey FA (1997) Strontium isotopic and trace element heterogeneity in the plains basalts of the Newer Volcanic Province, Victoria, Australia. Geochimica et Cosmochimica Acta. 10.1016/S0016-7037(96)00318-3

[CR75] Rajabi M, Tingay M, Heidbach O et al (2017) The present-day stress field of Australia. Earth-Sci Rev 168:165–189.

[CR76] Reynolds P, Brown RJ, Thordarson T et al (2015) Rootless cone eruption processes informed by dissected tephra deposits and conduits. Bull Volcanol. 10.1007/s00445-015-0958-3

[CR78] Rosengren NJ (1994) Eruption points of the Newer Volcanics Province of Victoria: an inventory and evaluation of scientific significance. National Trust of Australia (Victoria).

[CR79] Runge MG, Bebbington MS, Cronin SJ et al (2014) Vents to events: determining an eruption event record from volcanic vent structures for the Harrat Rahat, Saudi Arabia. Bull Volcanol 76:1–16. 10.1007/s00445-014-0804-z

[CR80] Ryan SM, Knight LA, Parker GJ (1995) The stratigraphy and structure of the Tyrendarra Embayment, Otway Basin, Victoria. Victorian Initiative for Minerals and Petroleum Report 15. Department of Agriculture, Energy and Minerals

[CR81] Sigmundsson F, Hooper A, Hreinsdóttir S et al (2015) Segmented lateral dyke growth in a rifting event at Bárðarbunga volcanic system, Iceland. Nature 517:191–195. 10.1038/nature14111 25517098 10.1038/nature14111

[CR82] Sigmundsson F, Parks M, Hooper A et al (2022) Deformation and seismicity decline before the 2021 Fagradalsfjall eruption. Nature 609:523–528. 10.1038/s41586-022-05083-4 36104559 10.1038/s41586-022-05083-4 PMC9477732

[CR83] Sigmundsson F, Parks M, Geirsson H et al (2024) Fracturing and tectonic stress drive ultrarapid magma flow into dikes. Science 283:1228–1235 (**(1979)**). 10.1126/science.adn2838 38330140

[CR84] Sigurdsson H (1968) Petrology of acid xenoliths from Surtsey. Geol Mag 105:440–453. 10.1017/S0016756800054820

[CR85] Smith IEM, Németh K (2017) Source to surface model of monogenetic volcanism: a critical review. In: Geological Society Special Publication. Geological Society of London, pp 1–28.

[CR86] Stroncik NA, Schmincke HU (2002) Palagonite - a review. Int J Earth Sci 91:680–697. 10.1007/s00531-001-0238-7

[CR87] Sun S -s., McDonough WF (1989) Chemical and isotopic systematics of oceanic basalts: implications for mantle composition and processes. In: Saunders AD, Norry MJ (eds) Magmatism in the Ocean Basins. Geological Society Special Publication, pp 313–345.

[CR88] Taddeucci J, Edmonds M, Houghton B, et al (2015) Hawaiian and strombolian eruptions. In: The Encyclopedia of Volcanoes. Elsevier, pp 485–503.

[CR89] Thordarson T, Larsen G (2007) Volcanism in Iceland in historical time: volcano types, eruption styles and eruptive history. J Geodyn 43:118–152. 10.1016/j.jog.2006.09.005

[CR90] Troll VR, Klügel A, Longpré M-A, et al (2011) Floating sandstones off El Hierro (Canary Islands, Spain): the peculiar case of the October 2011 eruption. Solid Earth Discussion 975–999. 10.5194/sed-3-975-2011

[CR91] Trowbridge R (2003) Palaeovolcanological processes of the Mt Eccles volcanic complex: a fissure vent system in the Newer Volcanics Province, Victoria, Australia. Masters thesis, Monash University, pp. 185

[CR92] Tsang SWR, Lindsay JM (2020) Lava flow crises in inhabited areas part I: lessons learned and research gaps related to effusive, basaltic eruptions. J Appl Volcanol. 10.1186/s13617-020-00096-y

[CR93] Tunjc JA (1988) The geology and geochemistry of the Mount Eccles volcanic complex, Western Victoria. BSc dissertation, LaTrobe University, pp. 93

[CR94] Valentine GA, Graettinger AH, Sonder I (2014) Explosion depths for phreatomagmatic eruptions. Geophys Res Lett 41:3045–3051. 10.1002/2014GL060096

[CR95] van den Hove JC, Grose L, Betts PG et al (2017a) Spatial analysis of an intra-plate basaltic volcanic field in a compressional tectonic setting: south-eastern Australia. J Volcanol Geoth Res 335:35–53. 10.1016/j.jvolgeores.2017.02.001

[CR96] van den Hove JC, Van Otterloo J, Betts PG et al (2017b) Controls on volcanism at intraplate basaltic volcanic fields. Earth Planet Sci Lett 459:36–47. 10.1016/j.epsl.2016.11.008

[CR97] van Otterloo J, Cas RAF, Sheard MJ (2013) Eruption processes and deposit characteristics at the monogenetic Mt. Gambier Volcanic Complex, SE Australia: implications for alternating magmatic and phreatomagmatic activity. Bull Volcanol 75:1–21. 10.1007/s00445-013-0737-y

[CR98] Van Otterloo J, Raveggi M, Cas RAF, Maas R (2014) Polymagmatic activity at the monogenetic Mt Gambier volcanic complex in the Newer Volcanics Province, SE Australia: new insights into the occurrence of intraplate volcanic activity in Australia. J Petrol 55:1317–1351. 10.1093/petrology/egu026

[CR99] Veevers JJ (1986) Breakup of Australia and Antarctica estimated as mid-Cretaceous (95 + 5 Ma) from magnetic and seismic data at the continental margin. Earth Planet Sci Lett. 10.1016/0012-821X(86)90135-4

[CR100] Vujovic A., Goldie Divoko LM, Eid R (2021) A review of structural elements, Otway Basin, Victoria. Victorian Gas Program Technical Report 64. Geological Survey of Victoria : Department of Jobs, Precincts and Regions, Melbourne

[CR101] Walker GPL (1973) Lengths of lava flows. Trans R Soc Lond Philos Trans Ser A Math Phys Eng Sci 274:107–118.

[CR102] Webb JA (2023) Western Victorian lava caves. pp 245–252.

[CR104] Wettenhall G (2010) The People of Budj Bim: engineers of aquaculture, builders of stone house settlements and warriors defending country. Em PRESS Publishing for the Gunditj Mirring Traditional Owners Aboriginal Corporation

[CR105] Wilkie B, Cahir F, Clark ID (2020) Volcanism in Aboriginal Australian oral traditions: ethnographic evidence from the Newer Volcanics Province. Journal of Volcanology and Geothermal Research. 10.1016/j.jvolgeores.2020.106999

[CR106] Wilson TM, Stewart C, Sword-Daniels V et al (2012) Volcanic ash impacts on critical infrastructure. Phys Chem Earth 45:5–23. 10.1016/j.pce.2011.06.006

[CR107] Yamamoto T, Soya T, Suto S et al (1991) The 1989 submarine eruption off eastern Izu Peninsula, Japan: ejecta and eruption mechanisms. Bull Volcanol 53:301–308.

[CR108] Ziesch J, Aruffo CM, Tanner DC et al (2017) Geological structure and kinematics of normal faults in the Otway Basin, Australia, based on quantitative analysis of 3-D seismic reflection data. Basin Res 29:129–148. 10.1111/bre.12146

