# Supplementary material for: Complex eruption processes and deposits of basaltic fissures: insights from the ~37 ka Budj Bim volcanic complex, Southeastern Australia
Source: Bull Volcanol. 2026 Mar 31;88(4):45. doi: 10.1007/s00445-026-01967-9 (PMC13038471; doi:10.1007/s00445-026-01967-9)
Supplement: Supplementary file 1 — (DOCX 30.6 MB) [file 445_2026_1967_MOESM1_ESM.docx]

## Supplementary Figures


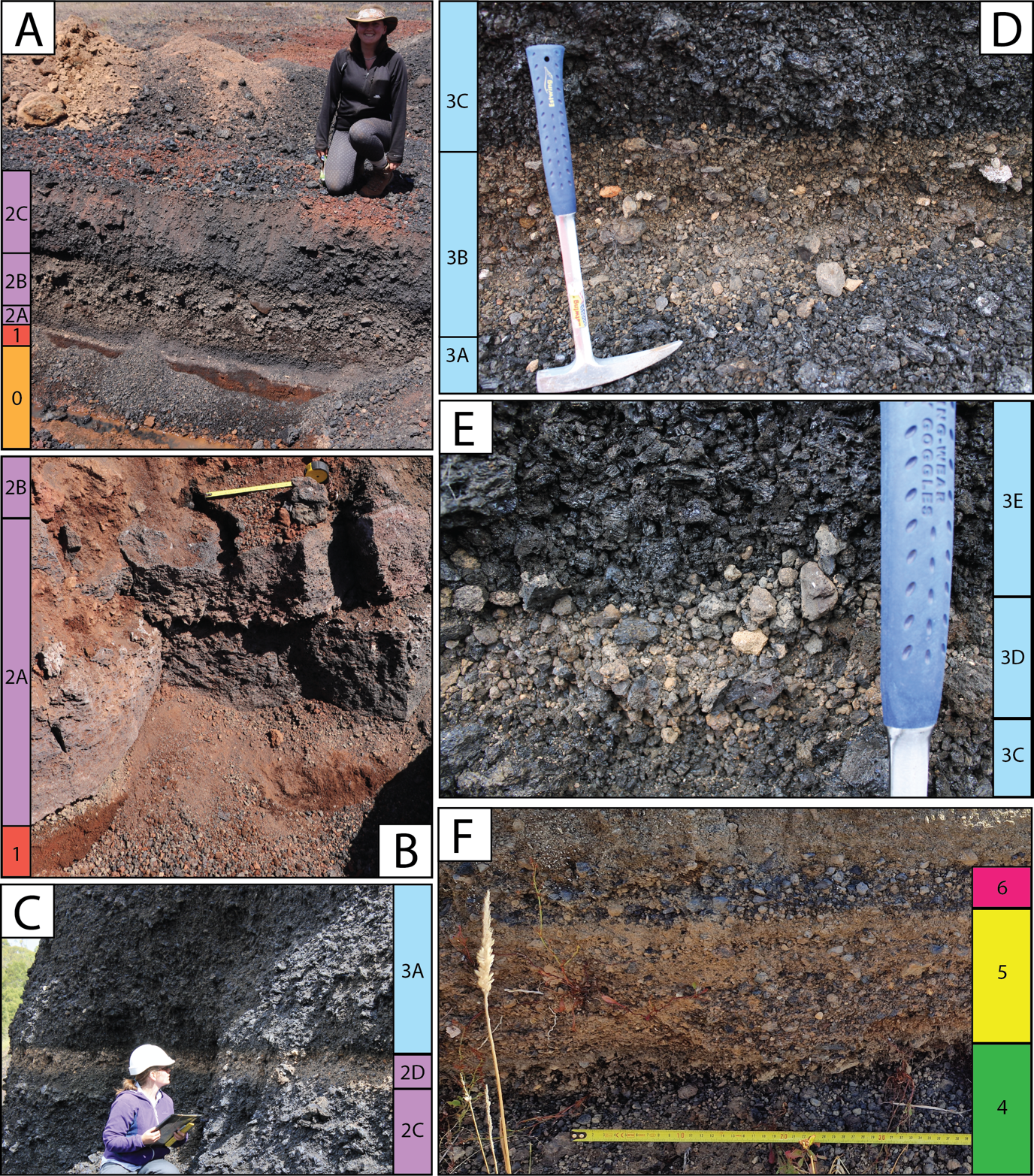


#### Figure S1: Little Mount Quarry deposits photographs with LM Units indicated: A) looking southeast (Locality BB16), showing lapilli scoria of Unit LM2A, B) looking south-southeast (Locality LM Lava Trench), showing lava flow of Unit LM2A, C) looking north (Locality 22), D) looking north (Locality 24), E) looking north (Locality 25), note the iridescence of basaltic scoria clasts within Unit LM3E, F) looking north (Locality ULM).


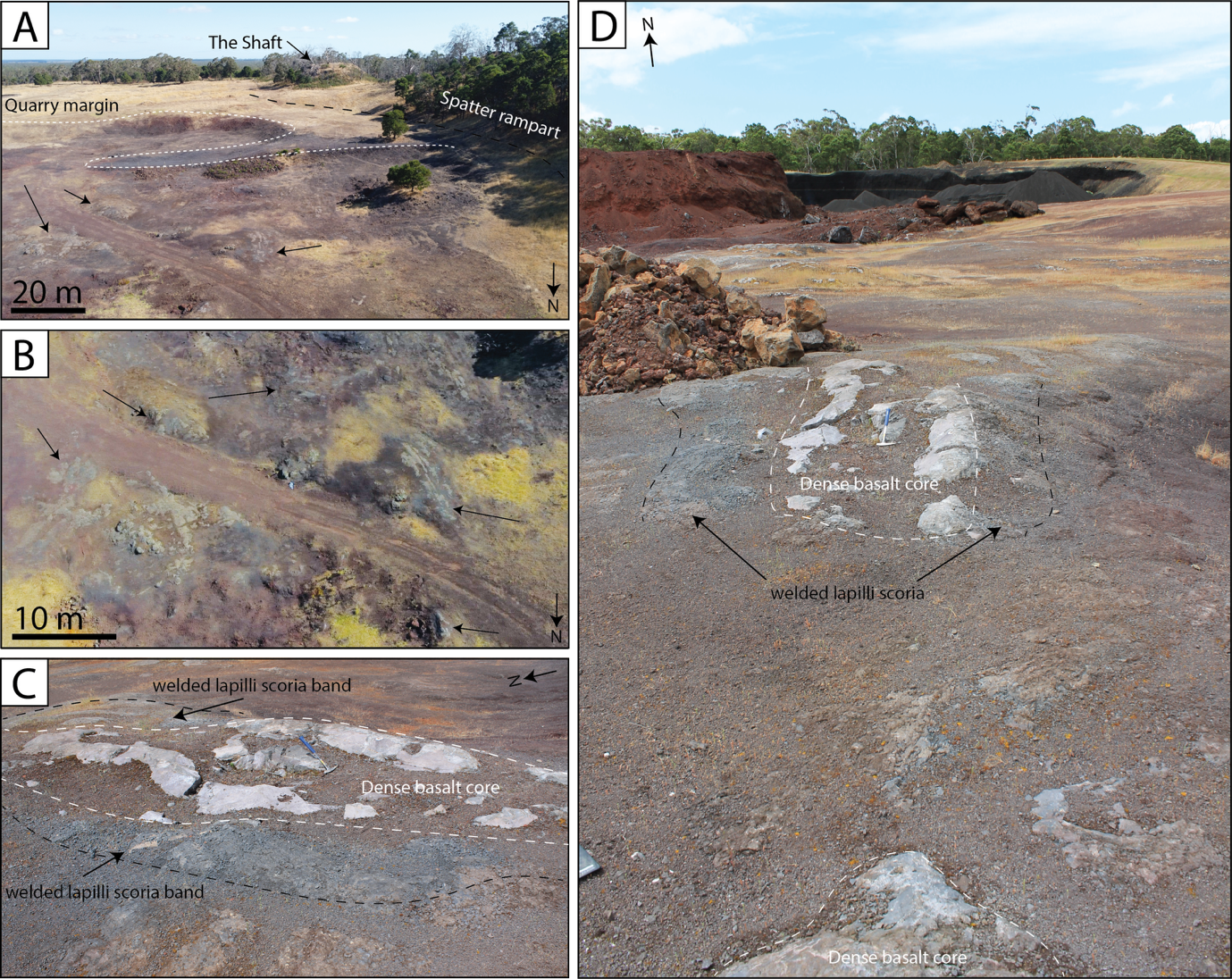


#### Figure S2: Feeder dyke segments exposed within the quarry floor at Little Mount: A-B) Drone photographs showing the location of coherent basalt mounds, C-D) outcrop photographs showing elongate dense basalt core (a basaltic dyke) in contact with welded lapilli scoria host material (40 cm long hammer for scale).


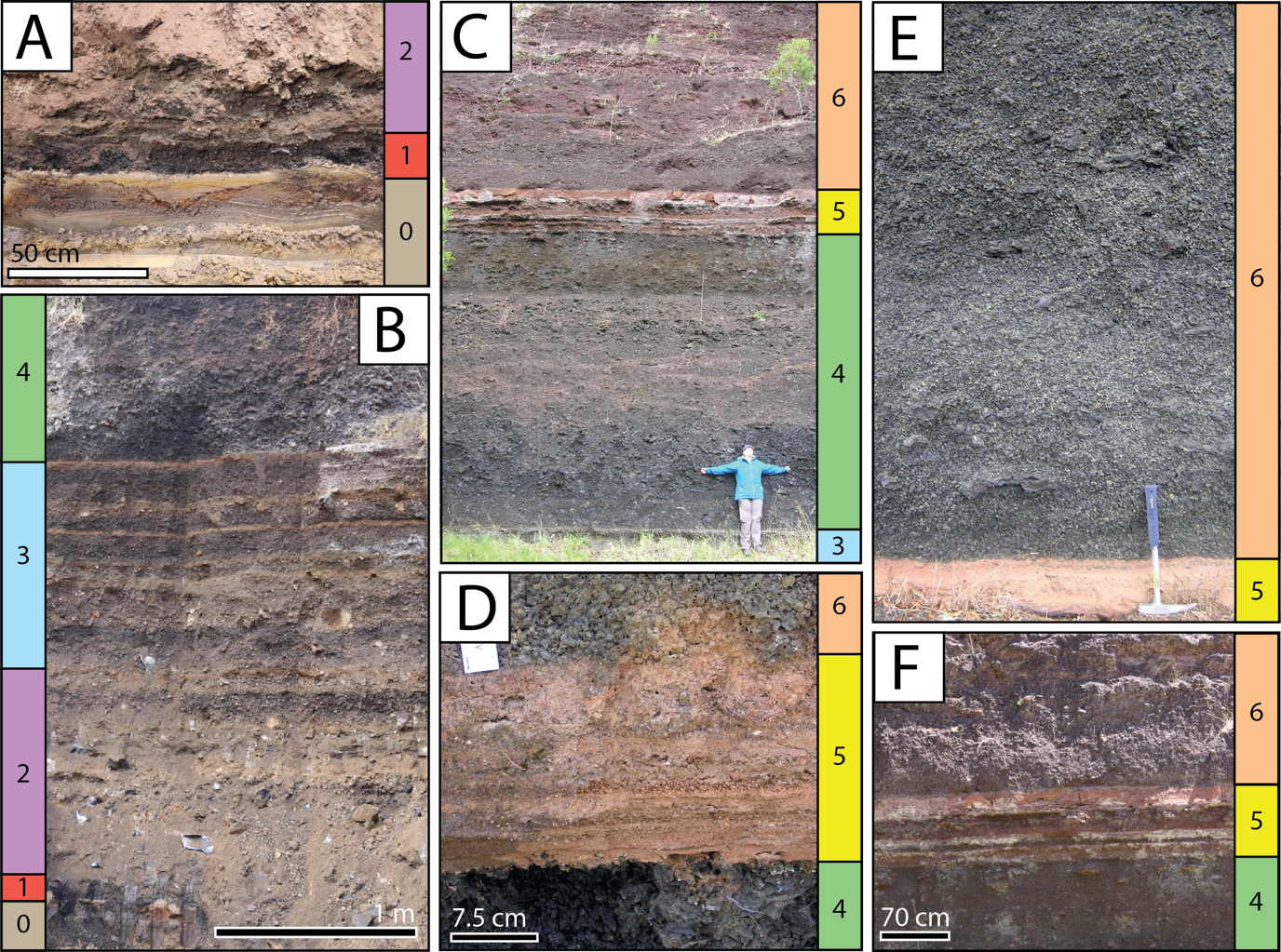


#### Figure S3: Porter’s Pit Quarry deposits photographs with PP stratigraphic units indicated, ordered from the top to the bottom of the exposure. A) cleaned face of the northwest cutting, showing contact with basal soil horizon, B) the northwest cutting, including basal soil horizon (unit 0), C) southwest cutting, looking towards the summit of Mount Eccles, D) detailed view of the southeast cutting (base of panel E), E) the southeast cutting, and F) the southwest cutting (close-up image of PP5 in panel C).


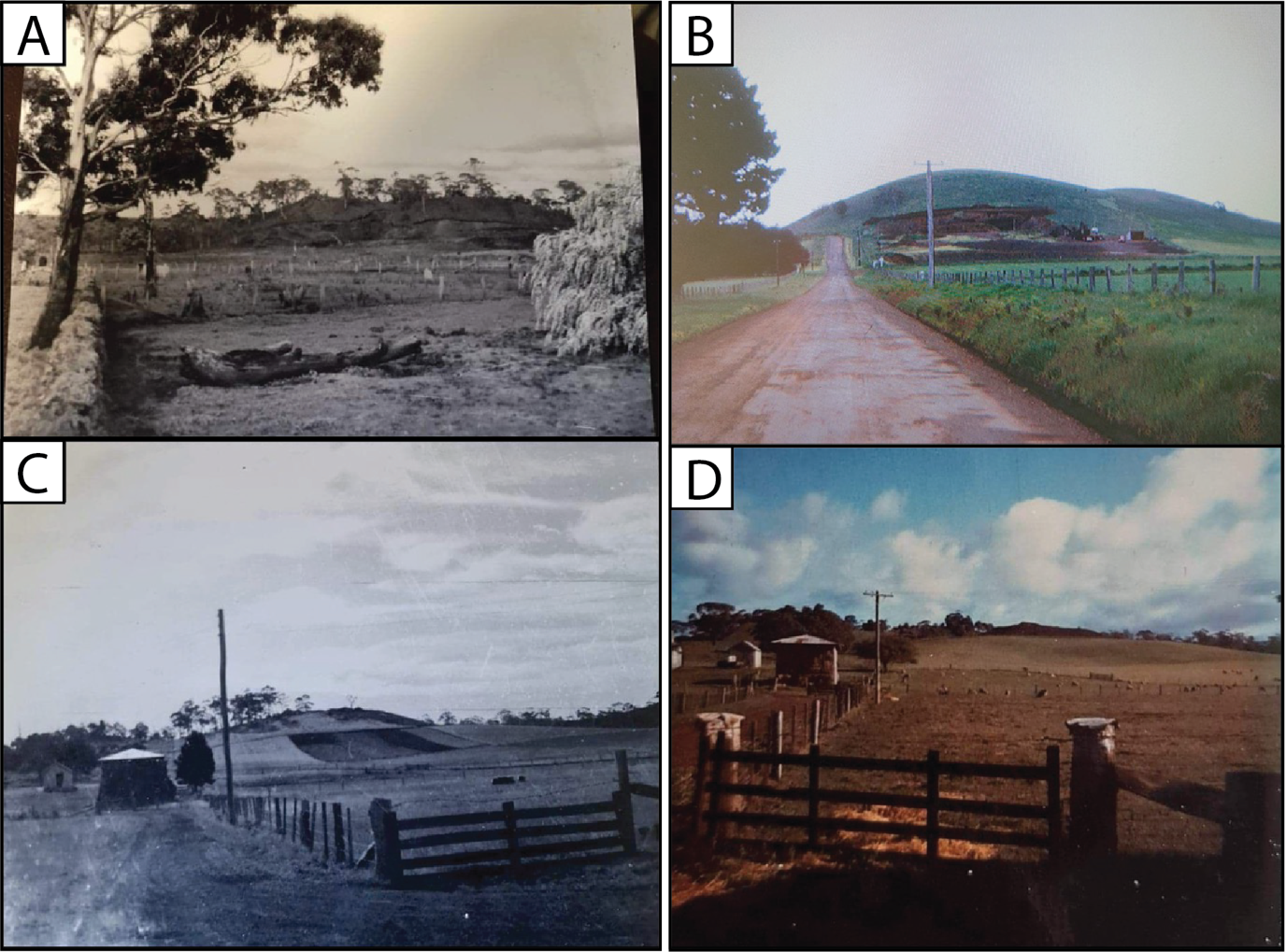


#### Figure S4: Photographs from the archive of Heather Rigby (c/o her son Mal Domaille, formerly of 64D Addinsall Rd and Douglas Addinsall’s nephew): A) View overlooking the volcanic features across farmland (photograph taken between 1952 and 1962), B) early development of the Porter’s Pit quarrying into the Mount Eccles cone, viewed looking west from the approach to Mount Eccles (photograph taken 1968), C) showing the original morphology ‘Little Mount’ scoria cone, looking west and showing the beginnings of quarrying into its eastern flank (photograph date unknown), and D) photograph taken from the same view point a panel C but years later, noting that the hill is largely absent having been quarried away.


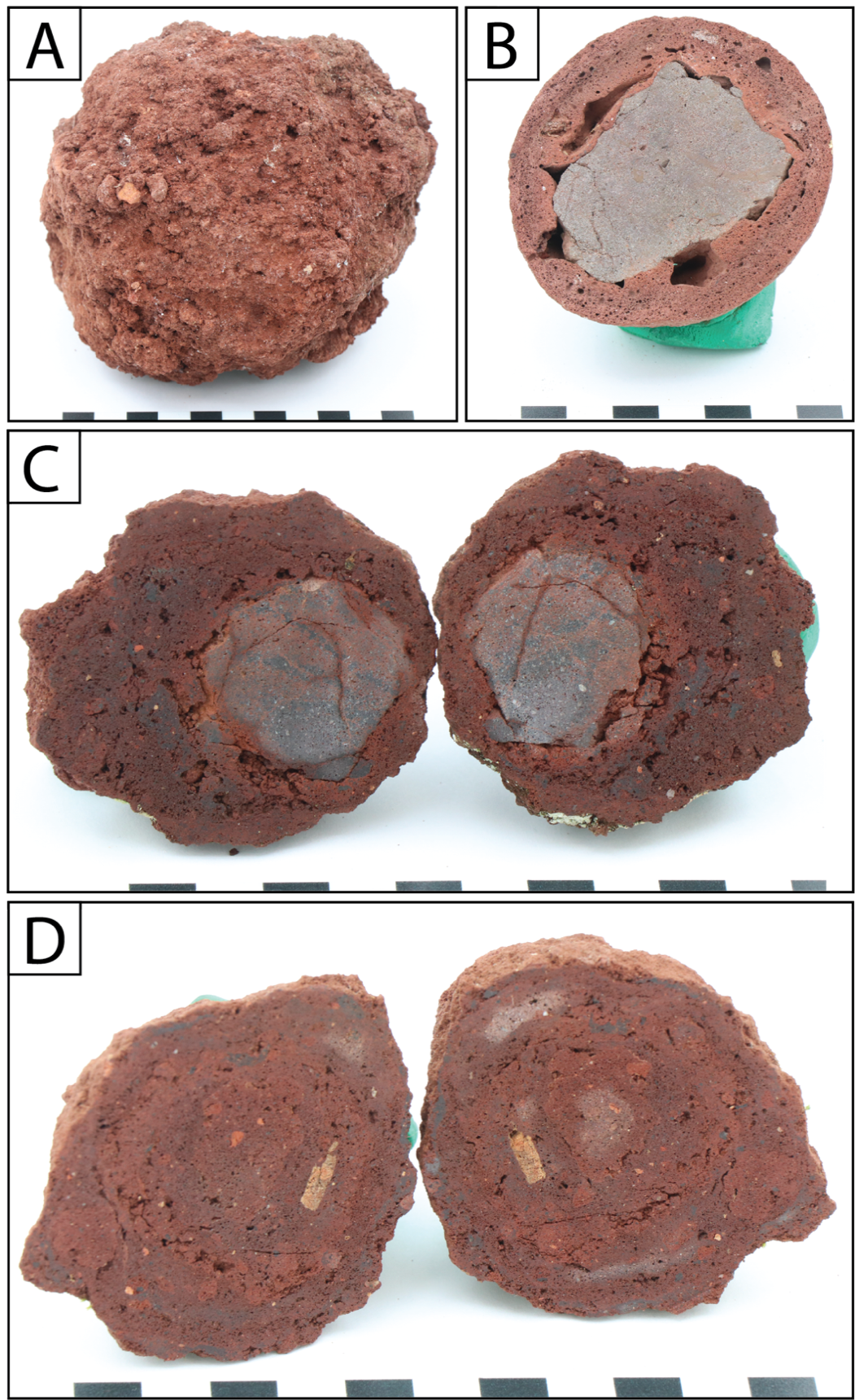


#### Figure S5: Armoured lapilli and bombs found within spatter rampart deposits near Little Mount Dry Crater: A) Typical red weathered sub-spherical morphology, B) a sample sliced open to reveal dense lava angular lithic core and vesicular juvenile lava rim with some large gas pockets, C) a sample sliced open to reveal angular, dense, porphyritic lava lithic core and vesicular basalt rim with minor sub-mm angular lithics (sample BBSE1a), and D) a sample cut open to reveal (possibly three) cm-thick rims of alternating vesicular/dense basalt with occasional cm-size macrocrysts (sample BBJH4). A centimetre-scale bar is included in all photographs.


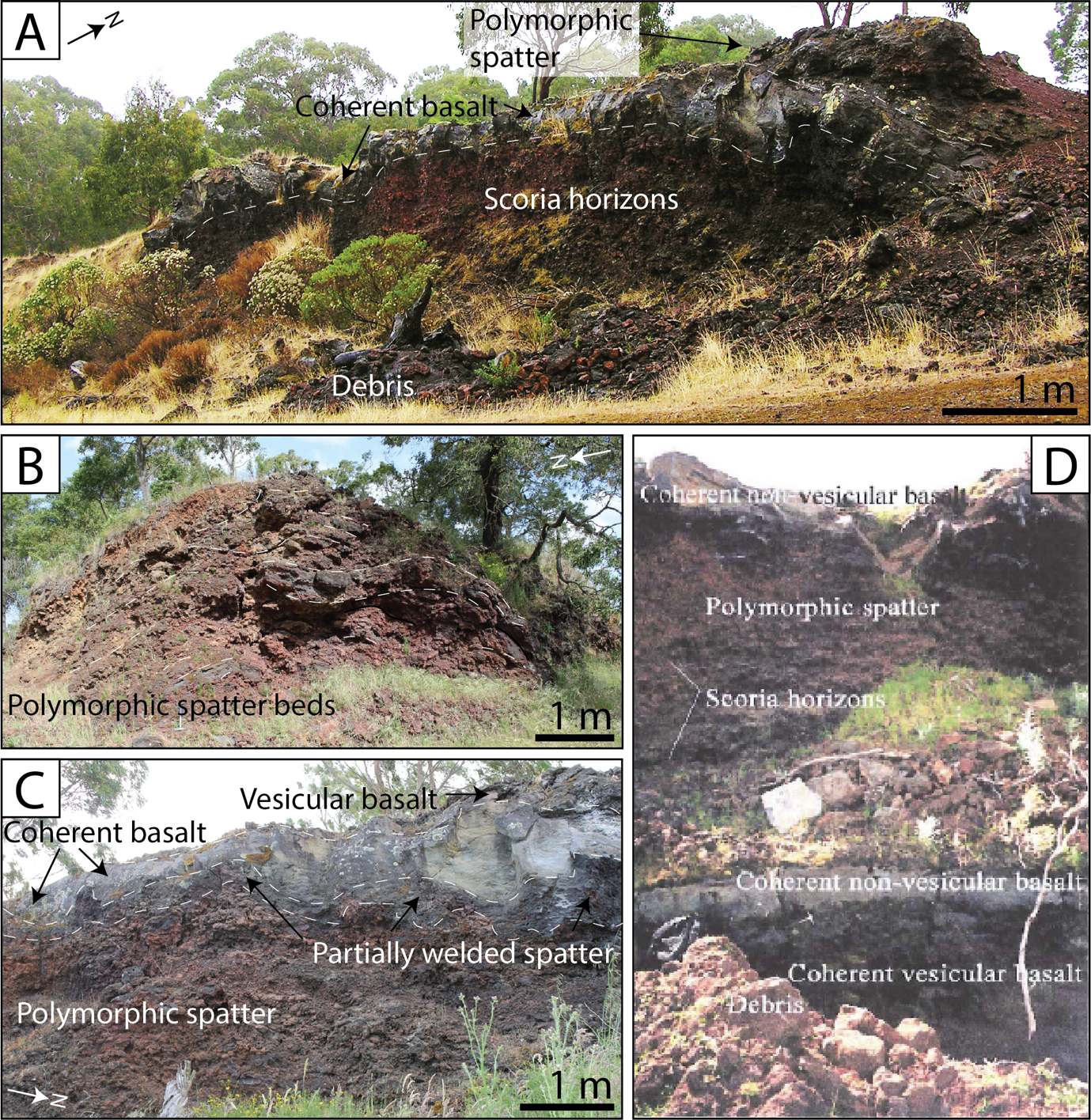


#### Figure S6: Addinsalls Pit lava and spatter deposits: A) lava and spatter exposed to the west of the entrance to the quarry, B) arcuate spatter beds creating a spatter mound to the east of the pit entrance, C) close-up photograph of welded spatter beds topped by thick, dense basalt lava flow to the west of the quarry entrance, and D) archive photograph from Trowbridge (2003) showing coherent non-vesicular and coherent vesicular basalt at the base of the sequence (currently hidden beneath debris indicated in panel A).

#### **
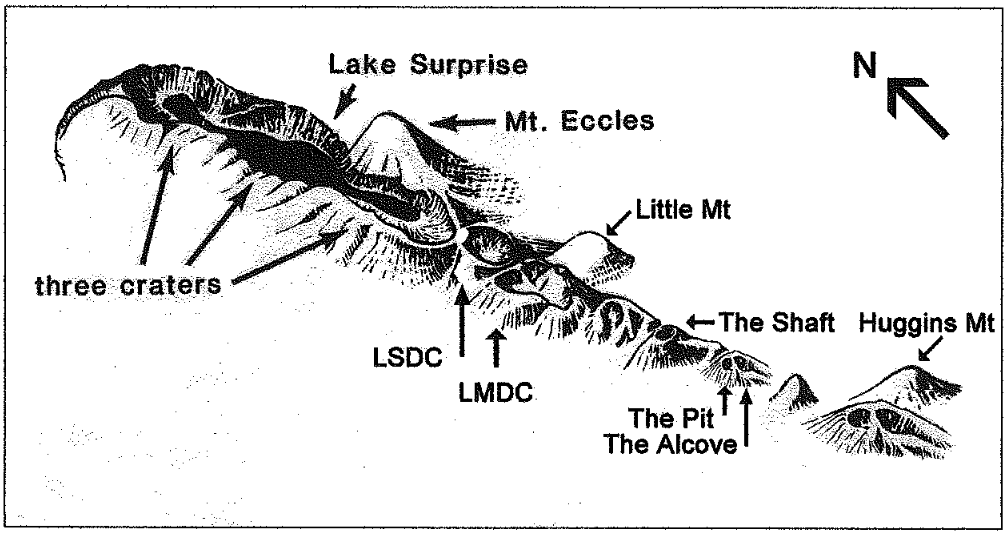
**Figure S7: Schematic representation of the crater and cones of the BBVC and the key named structures (Trowbridge, 2003; modified after the Mt Eccles National Park visitor guide, 2001).


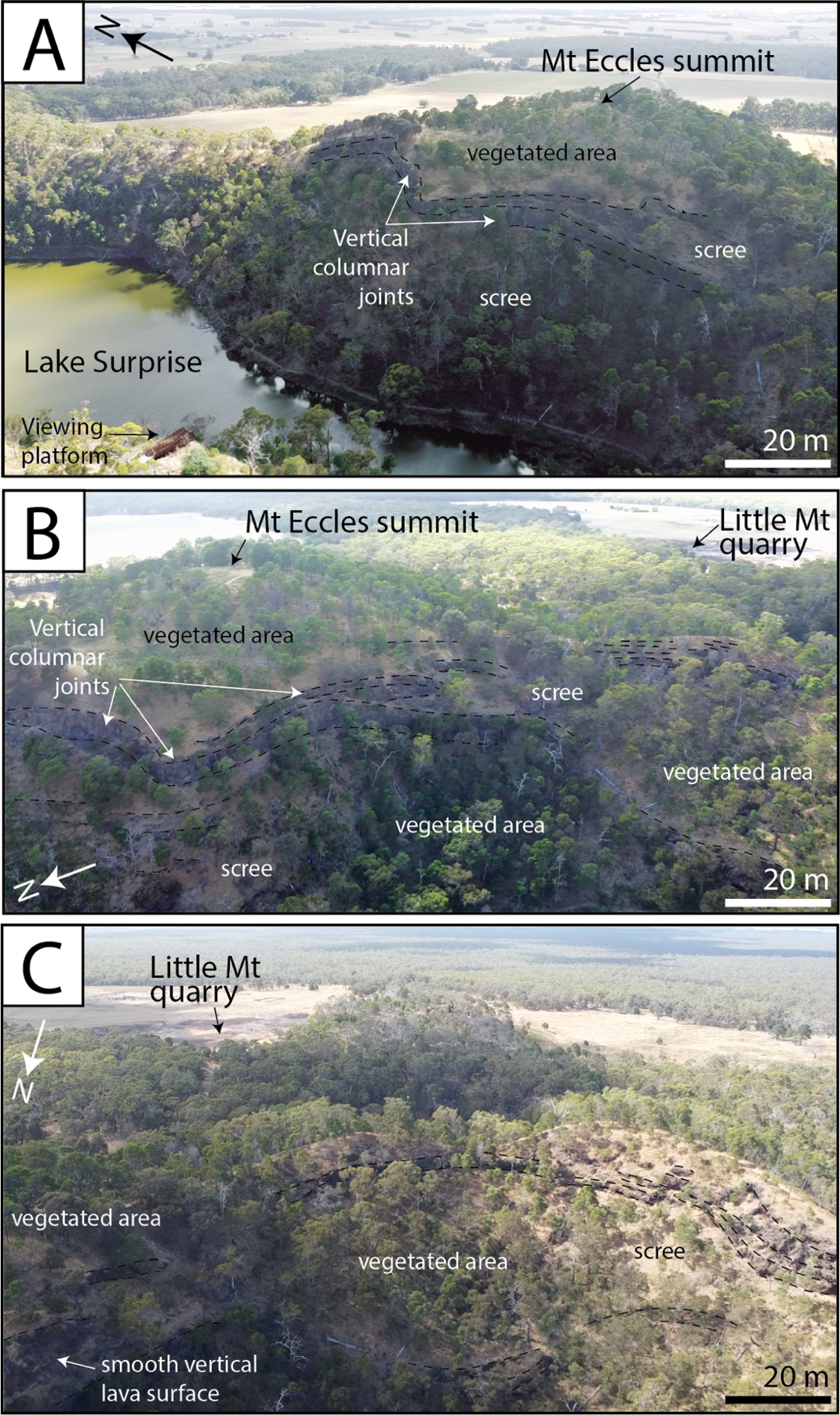


#### Figure S8: Overview drone photographs of the Lake Surprise crater showing lava flow boundaries (dashed line) and noting the occurrence of columnar joints in the thickest flows and rare surfaces of smooth vertical lava. Viewed looking northeast (A), southeast (B) and south-southeast (C); the scale bar is indicative for the foreground only.


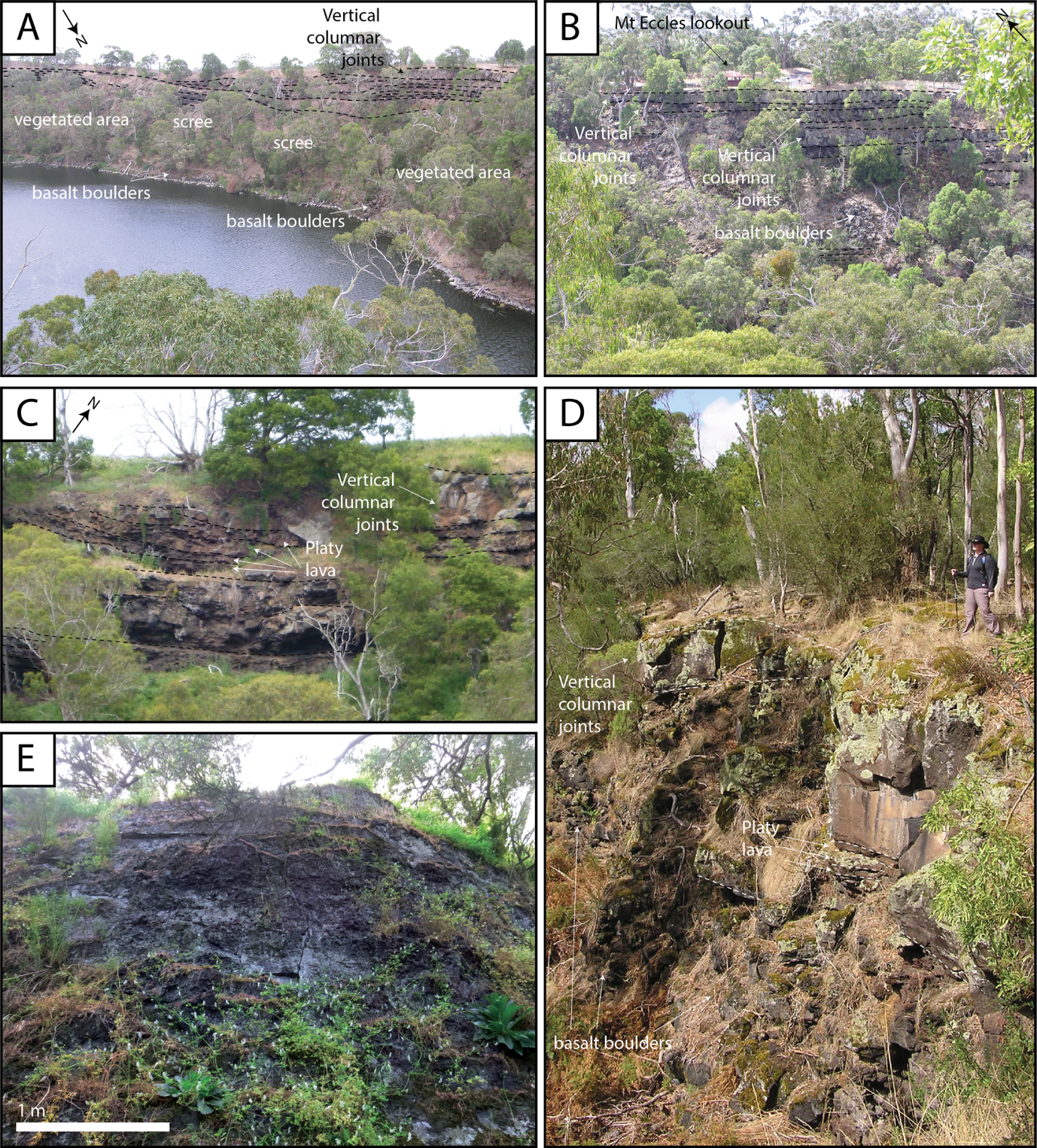


#### Figure S9: Lake Surprise fissure crater internal surfaces and structure. Views of the steep scree-covered crater walls which rise ~20 m above the lake surface: A) looking south-southwest, B) looking northeast and C) looking northwest. Occasional columnar-jointed lava flows at the highest exposures overly thin undulous lava flows with irregular contacts (black dashed lines indicate approximate boundaries between some flow units). Lose basalt lava boulders created by post-eruption erosion have collected at the base of the walls and at the lake margin. D) Alternating flat-lying columnar-jointed coherent lava flows with incoherent, agglutinated lava and spatter horizons (white dashed lines indicate approximate boundaries between flow units). E) Smooth and near-vertical lava-coated downward dipping crater surface (79º dip) exposed within the crater.


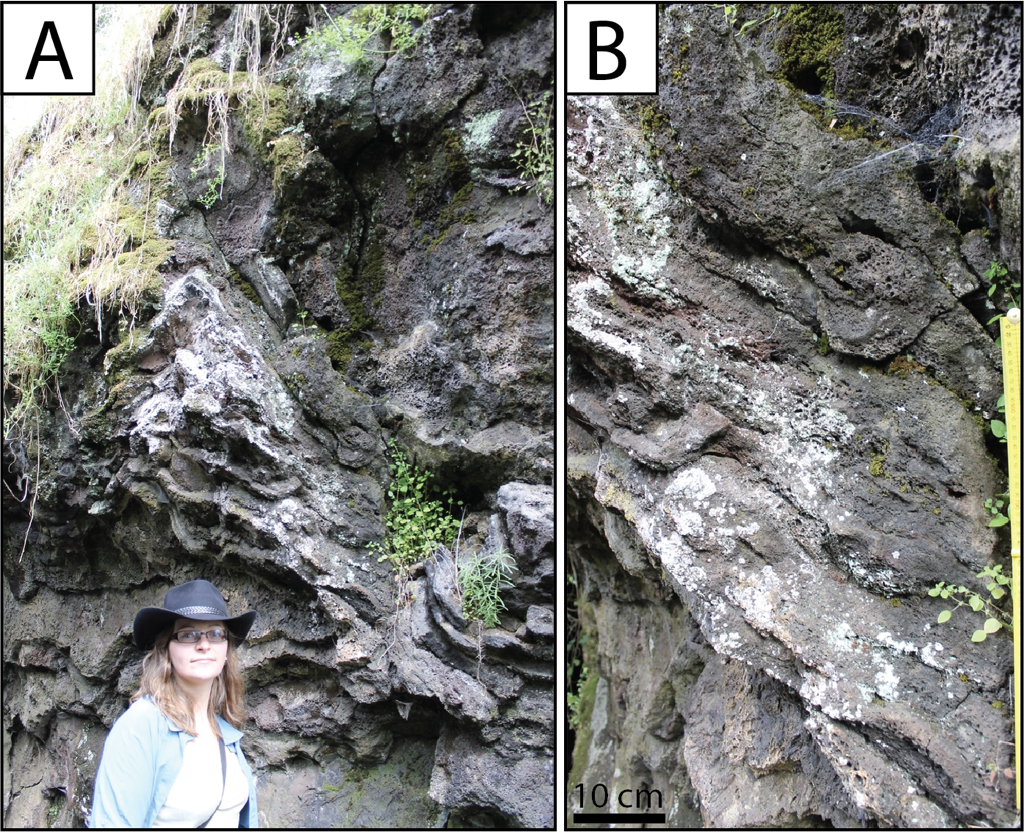


#### Figure S10: Lava flows of the BBVC at Natural Bridge: A) steep and thin lava sheet morphology of lava levees near Natural Bridge, and B) close-up photograph of A) showing steeply dipping, variably vesiculated beds.
