# Supplementary material for: Complex eruption processes and deposits of basaltic fissures: insights from the ~37 ka Budj Bim volcanic complex, Southeastern Australia
Source: Bull Volcanol. 2026 Mar 31;88(4):45. doi: 10.1007/s00445-026-01967-9 (PMC13038471; doi:10.1007/s00445-026-01967-9)
Supplement: Supplementary file 2 — (DOCX 28.1 KB) [file 445_2026_1967_MOESM2_ESM.docx]

#### Table S1: Samples selected for thin sectioning and geochemistry, ordered by Site (from north to south: the lava flow field (north, central and south), the Lake Surprise volcanic fissure crater, Porter’s Pit Quarry, the Little Mount Quarry, The Pit, The Shaft and Addinsalls Pit), noting initial rock classification (where M/scoria = mingled scoria). Eruptive Units refer to Tables 2 (LM) and 3 (PP), (+) indicates repeat measurement of archive sample from Trowbridge (2003). Weathering of samples: F = Fresh, S = slightly weathered, W = weathered, V = very weathered. Latitude (º) and Longitude (º) of collection site and type of analysis conducted (petrographic analysis of thin sections, XRF Major and Trace Elements analysed, and Radiogenic Isotopes measured.

| Sample | Date Collected | Site Name | Classification | Eruptive Unit | Weathering | Lat (º) | Lon (º) | Pet. Analysis | XRF Maj. | XRF Tr. | Radiogenic Isotopes |
| --- | --- | --- | --- | --- | --- | --- | --- | --- | --- | --- | --- |
| MEC19 | 03/03/2012 | Lava Field (N) | Lava |  | F | -38.0222 | 141.8481 | * | * | * | * |
| MEC31 | 06/11/2012 | Lava Field (C) | Lava |  | F | -38.0757 | 141.9051 |  | * | * |  |
| MEC27 | 06/11/2012 | Lava Field (S) | Lava |  | F | -38.1106 | 141.9012 | * | * | * |  |
| MEC33 | 06/11/2012 | Lake Surprise | Lava |  | S | -38.0587 | 141.9215 | * | * | * | * |
| 39A(+) | 2002-2003 | PP Quarry | Scoria | PP4 | S | -38.0633 | 141.9261 |  | * |  |  |
| 55F(+) | 2002-2003 | PP Quarry | Scoria | PP6+ | S | -38.0633 | 141.9261 |  | * |  |  |
| MEC4 | 02/03/2012 | PP Quarry | Scoria | PP2 | W | -38.0611 | 141.9282 |  | * | * |  |
| MEC7 | 02/03/2012 | PP Quarry | Scoria | PP4 | F | -38.0610 | 141.9285 |  | * |  |  |
| MEC7A | 02/03/2012 | PP Quarry | Scoria | PP4 | F | -38.0610 | 141.9285 |  | * | * | * |
| MEC7B | 02/03/2012 | PP Quarry | Spatter | PP4 | F | -38.0610 | 141.9285 | * | * | * |  |
| MEC9 | 02/03/2012 | PP Quarry | Scoria | PP6 | S | -38.0601 | 141.9285 |  | * | * |  |
| MEC45 | 08/11/2012 | LM Quarry | Dyke | Dyke | F | -38.0678 | 141.9293 | * | * | * |  |
| MEC47A | 08/11/2012 | LM Quarry | M/scoria |  | F | -38.0664 | 141.9299 | * |  |  |  |
| MEC49 | 08/11/2012 | LM Quarry | Scoria | LM3A | F | -38.0664 | 141.9299 |  | * | * | * |
| MEC49Ai | 08/11/2012 | LM Quarry | M/scoria |  | F | -38.0664 | 141.9299 | * |  |  |  |
| MEC49Aii | 08/11/2012 | LM Quarry | M/scoria |  | F | -38.0664 | 141.9299 | * |  |  |  |
| MEC49C | 08/11/2012 | LM Quarry | M/scoria | LM3A | F | -38.0664 | 141.9299 | * | * |  |  |
| MEC51 | 08/11/2012 | LM Quarry | Lava |  | F | -38.0669 | 141.9299 | * | * | * |  |
| MEC53 | 09/11/2012 | LM Quarry | Scoria | LM3C | F | -38.0668 | 141.9305 | * | * | * |  |
| MEC53D | 09/11/2012 | LM Quarry | Lithic | LM3C | W | -38.0669 | 141.9305 |  | * |  |  |
| MEC55 | 09/11/2012 | LM Quarry | Scoria | LM3E | F | -38.0669 | 141.9305 |  | * | * |  |
| MEC55B | 09/11/2012 | LM Quarry | Scoria | LM3B | F | -38.0669 | 141.9305 |  | * | * |  |
| MEC59 | 09/11/2012 | The Pit | Lava |  | F | -38.0711 | 141.9295 | * | * | * | * |
| MEC57 | 09/11/2012 | The Shaft | Lava |  | V | -38.0697 | 141.9307 |  | * | * |  |
| 53(+) | 2002-2003 | Addn Pit | Spatter | + | F | -38.0763 | 141.9315 |  | * |  |  |
| MEC17 | 03/03/2012 | Addn Pit | Spatter |  | S | -38.07278 | 141.9312 | * | * | * |  |
| MEC18 | 03/03/2012 | Addn Pit | Lava |  | F | -38.07278 | 141.9312 | * | * | * | * |
| MEC64A | 09/11/2012 | Addn Pit | Lava |  | F | -38.073 | 141.9311 |  | * | * |  |
