# Supplementary material for: Complex eruption processes and deposits of basaltic fissures: insights from the ~37 ka Budj Bim volcanic complex, Southeastern Australia
Source: Bull Volcanol. 2026 Mar 31;88(4):45. doi: 10.1007/s00445-026-01967-9 (PMC13038471; doi:10.1007/s00445-026-01967-9)
Supplement: Supplementary file 3 — (DOCX 28.1 KB) [file 445_2026_1967_MOESM3_ESM.docx]

#### Table S2: Geographic locations of main volcanic structures, sampling and logging localities.

| General location | Latitude | Longitude |
| --- | --- | --- |
| Porter’s Pit Quarry | 38.0611 S | 141.9281 E |
| Little Mount Quarry | 38.0664 S | 141.9299 E |
| The Pit | 38.0711 S | 141.9295 E |
| The Shaft | 38.0697 S | 141.9307 E |
| Addinsalls Pit Quarry | 38.0727 S | 141.9312 E |
| Lake Surprise | 38.0587 S | 141.9215 E |
