# Supplementary material for: Complex eruption processes and deposits of basaltic fissures: insights from the ~37 ka Budj Bim volcanic complex, Southeastern Australia
Source: Bull Volcanol. 2026 Mar 31;88(4):45. doi: 10.1007/s00445-026-01967-9 (PMC13038471; doi:10.1007/s00445-026-01967-9)
Supplement: Supplementary file 5 — (DOCX 75.0 KB) [file 445_2026_1967_MOESM5_ESM.docx]

## Supplementary Text

### Crystal fabric analysis method

To assess the extent of plagioclase alignment within samples, a Nikon Eclipse LV100N POL microscope was used to collect photomicrographs in plane polarised light (PPL) and cross polarised light (XPL) at six different locations within the slide. Each XPL photomicrograph was then processed with the software JMicrovision (version 1.3.4) and plagioclase crystals of the groundmass were isolated by enhancing the contrast, converting each image to binary, and automatically tracing the contour of the crystals. A surface filter was set to exclude crystals larger than 10,000 μm^2^ from the analysis as microlites are defined as crystals less than 100 microns in length (Mangler et al. 2022). After this processing, each image was checked and any residual noise occurring after the automatic tracing of the crystal’s borders removed manually. The length, width, orientation (with respect to the long edge of the section) and area of individual crystals were determined (Roduit 2008; Payacán et al. 2014). These data were then exported and processed using MATLAB to plot a unidirectional rose diagram of orientations and a histogram of grain area.

### Geochemical analysis methods

Lava samples were sliced using a diamond blade to remove any evidence of external weathering. Scoria and spatter clasts were screened for visible signs of weathering, cleaned in an ultrasonic bath filled with distilled water, with the water changed at 1-minute intervals, if necessary, until the water was clear. The cleaned samples were then dried in an oven at 90 ºC, crushed using a rock splitter and jaw crusher, and again screened for weathered material. The cleaned rock chips were then pulverised to a powder in a tungsten carbide mill – because of the potential for contamination, Ta and Co are not used here.

Whole-rock geochemistry was measured for major (25 samples) and trace (18 samples) elements using X-ray Fluorescence spectroscopy (XRF) at James Cook University (JCU), Australia and the University of Leicester (UoL), UK. At JCU an aliquot of powder from each sample was ignited at 1000 ºC for 4 hours for Loss on Ignition and then fused discs were prepared using a Li-metaborate-tetraborate flux at 1050-1100 ºC, with measurements made with a Bruker-AXS S4 Pioneer operated by Spectra Plus software. At UoL, major elements were determined on fused glass beads prepared from ignited powders with a sample to flux ratio 1:5 (80% Li metaborate: 20% Li tetraborate flux). Trace elements were analysed on 32 mm diameter pressed powder pellets produced from mixing 7 g of fine ground sample with 12-15 drops of a 7% PVA solution (Moviol 8-88) and pressed at 10 tonnes per square inch. The samples were then analysed using a PANalytical Axios Advanced XRF spectrometer. At both laboratories, typical precision for major elements is better than 1% (1SD) although no secondary standards were run within the analyses. Following analysis, the total Fe_2_O_3_ reported was revised to include ferrous iron following a 3:17 ferric:ferrous iron ratio (Middlemost 1989).

Based on the whole-rock XRF results, a subset of six samples were selected for Sr-Nd-Pb isotope analysis at the University of Melbourne, using a NU Plasma MC–ICPMS and following the modified method of Maas et al. (2005) described by Van Otterloo et al. (2014). 100-150 mg of the selected samples were weighed out and then leached in acid (6M HCl) at 100 ºC for 30 minutes. These samples were then dissolved on a hotplate using a combination of concentrated HF and HNO_3_. Sr and Nd were extracted using EICHROM SR-, RE- and LN- resins. Blanks were negligible, at <0.1 ng. Isotopic analyses were carried out on a Nu Plasma multicollector (MC)-ICP-MS system. Nd data are normalised to ^146^Nd/^144^Nd = 0.7219 and are reported relative to La Jolla Nd = 0.511860. External precision (2SD) is <± 0.000020. Analyses of the BCR-2 and BHVO-2 rock reference materials run with the unknown samples gave a value of 0.512648 ± 0.000008 (2σ) and 0.513003 ± 0.000006 (2σ), respectively, compared with published values of 0.512634 ± 0.000012 and 0.512979 ± 0.000014 (Weis et al. 2006; Jochum et al. 2007). Sr data were normalised to ^86^Sr/^88^Sr = 0.1194 and are reported relative to SRM987 Sr = 0.710230. External precision (2SD) is <± 0.000040. Analyses of the BCR-2 and BHVO-2 rock reference materials run with the unknown samples gave a value of 0.704989 ± 0.000021 (2σ) and 0.703453 ± 0.000021 (2σ), respectively, compared with published values of 0.705019 ± 0.000016 and 0.703487 ± 0.000019 (Weis et al. 2006). Pb mass bias was corrected using thallium doping and a ^205^Tl/^203^Tl ratio of 2.3871. For signal sizes near 10 V total Pb, this produces internal precisions of ≤±0.01% (2SE) and external precisions of 0.025–0.05% (2SD) in ^206,207,208^Pb/^204^Pb ratios. Three runs of SRM981-Pb yielded the following averages: ^206^Pb/^204^Pb 16.931 ± 1, ^207^Pb/^204^Pb 15.485 ± 2, ^208^Pb/^204^Pb 36.694 ± 9 (all errors 2SD). All results compare well with thermal ionization mass spectrometry and MC-ICP-MS reference values (e.g. Doucelance and Manhes 2001; Baker et al. 2004).

## Supplementary References

Baker J, Peate D, Waight T, Meyzen C (2004) Pb isotopic analysis of standards and samples using a 207Pb-204Pb double spike and thallium to correct for mass bias with a double-focusing MC-ICP-MS. Chem Geol 211:275–303. https://doi.org/10.1016/j.chemgeo.2004.06.030

Doucelance R, Manhes G (2001) Reevaluation of precise lead isotope measurements by thermal ionization mass spectrometry: comparison with determinations by plasma source mass spectrometry. Chem Geol 176:361–377

Jochum KP, Stoll B, Herwig K, Willbold M (2007) Validation of LA-ICP-MS trace element analysis of geological glasses using a new solid-state 193 nm Nd:YAG laser and matrix-matched calibration. J Anal At Spectrom 22:112–121. https://doi.org/10.1039/b609547j

Maas R, Kamenetsky MB, Sobolev A V., et al (2005) Sr, Nd, and Pb isotope evidence for a mantle origin of alkali chlorides and carbonates in the Udachnaya kimberlite, Siberia. Geology 33:549–552. https://doi.org/10.1130/G21257.1

Mangler MF, Humphreys MCS, Wadsworth FB, et al (2022) Variation of plagioclase shape with size in intermediate magmas: a window into incipient plagioclase crystallisation. Contributions to Mineralogy and Petrology 177:. https://doi.org/10.1007/s00410-022-01922-9

Middlemost EAK (1989) Iron oxidation ratios, norms and the classification of volcanic rocks. Chem Geol 77:19–26

Payacán I, Gutiérrez F, Gelman SE, et al (2014) Comparing magnetic and magmatic fabrics to constrain the magma flow record in La Gloria pluton, central Chile. J Struct Geol 69:32–46. https://doi.org/10.1016/j.jsg.2014.09.015

Roduit N (2008) JMicroVision: Image Analysis Toolbox for Measuring and Quantifying Components of High-definition Images

Van Otterloo J, Raveggi M, Cas RAF, Maas R (2014) Polymagmatic activity at the monogenetic Mt Gambier volcanic complex in the Newer Volcanics Province, SE Australia: New insights into the occurrence of intraplate volcanic activity in Australia. Journal of Petrology 55:1317–1351. https://doi.org/10.1093/petrology/egu026

Weis D, Kieffer B, Maerschalk C, et al (2006) High-precision isotopic characterization of USGS reference materials by TIMS and MC-ICP-MS. Geochemistry, Geophysics, Geosystems 7:. https://doi.org/10.1029/2006GC001283
